# Smart and versatile biomaterials for cutaneous wound healing

**DOI:** 10.1186/s40824-023-00426-2

**Published:** 2023-09-16

**Authors:** Minxiong Li, Wenzheng Xia, Yi Min Khoong, Lujia Huang, Xin Huang, Hsin Liang, Yun Zhao, Jiayi Mao, Haijun Yu, Tao Zan

**Affiliations:** 1 Department of Plastic and Reconstructive Surgery, Shanghai Ninth People’s Hospital, Shanghai Jiao Tong University School of Medicine, Shanghai, 200011 China; 2 Center of Pharmaceutics, Shanghai Institute of Materia Medica, Chinese Academy of Sciences, Shanghai, 201203 China

**Keywords:** Wound healing, Smart dressings, Stimuli-responsive, Biomaterials

## Abstract

The global increase of cutaneous wounds imposes huge health and financial burdens on patients and society. Despite improved wound healing outcomes, conventional wound dressings are far from ideal, owing to the complex healing process. Smart wound dressings, which are sensitive to or interact with changes in wound condition or environment, have been proposed as appealing therapeutic platforms to effectively facilitate wound healing. In this review, the wound healing processes and features of existing biomaterials are firstly introduced, followed by summarizing the mechanisms of smart responsive materials. Afterwards, recent advances and designs in smart and versatile materials of extensive applications for cutaneous wound healing were submarined. Finally, clinical progresses, challenges and future perspectives of the smart wound dressing are discussed. Overall, by mapping the composition and intrinsic structure of smart responsive materials to their individual needs of cutaneous wounds, with particular attention to the responsive mechanisms, this review is promising to advance further progress in designing smart responsive materials for wounds and drive clinical translation.

**Graphical Abstract:**

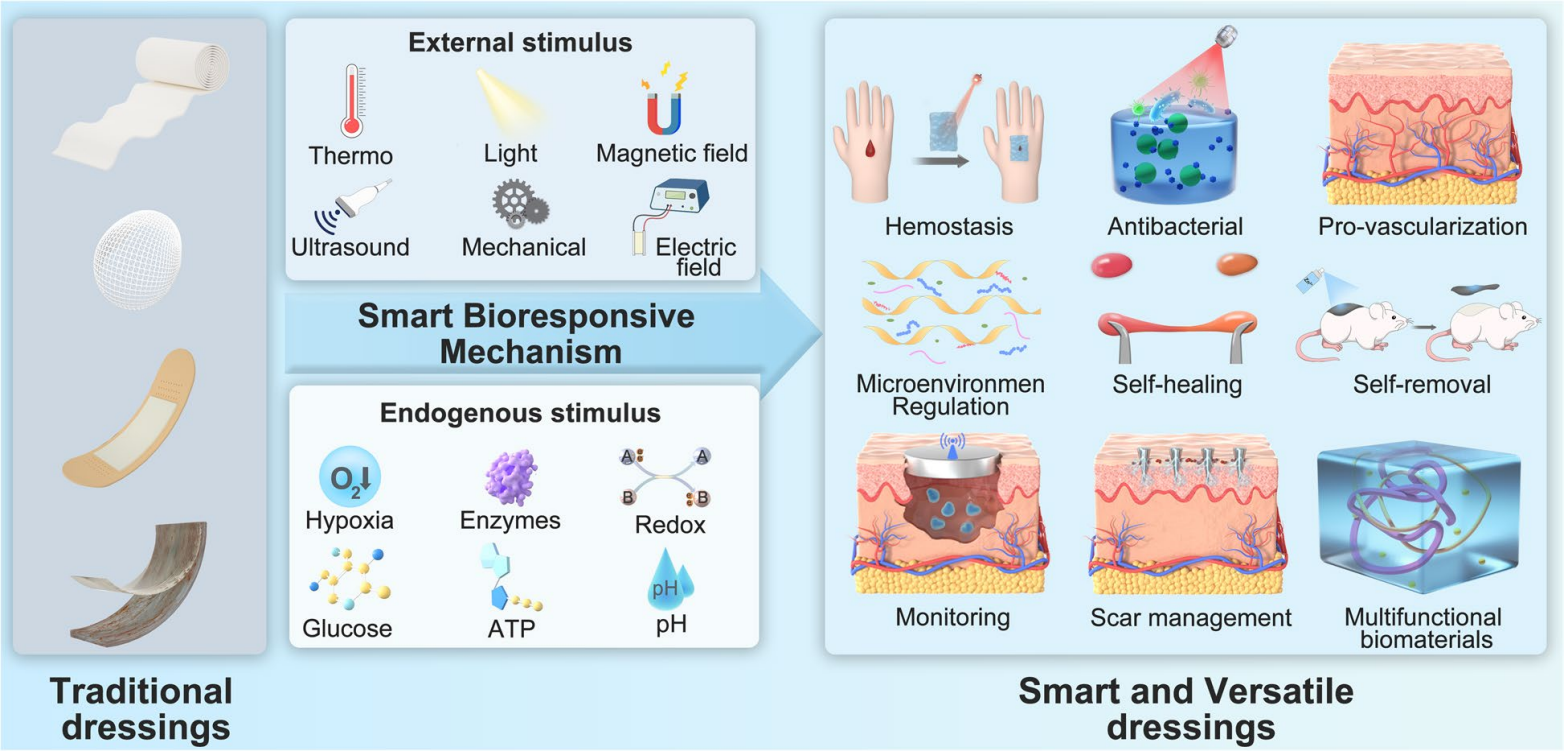

## Introduction

As the largest organ of the body, the skin functions as a barrier to protect internal organs from environmental threats [1]. Resulting from various conditions (e.g., trauma, burns, chronic diseases, tumor resection, etc.), cutaneous wounds often induce a sequence of complications (such as tissue damage, organ failure and septicemia), putting patients’ lives in jeopardy [2]. Skin disorders are estimated to affect one-third of the global population, with wound management posing a huge health and financial burdens on patients and society [3]. In addition to the localized wound factors (wound depth, wound size and wound infection), the healing process can also be hampered by other factors (such as systematic immunological or nutritional deficiency, age, chronic co-morbidities, etc.) [4]. Despite intensive research to improve cutaneous wound healing, clinical wound treatment in many circumstances remains inadequate.

Wound dressing has been used for millennia to protect wounds from injury and promote healing [5]. Covering wounds with leaves, cloth or natural ointments was one of the earliest treatments, with the goal of reducing pain, preventing infection, and hastening wound closure. Until now, a variety of wound dressings have been developed in clinical practice, including gauze, foams, hydrogels and other materials [6]. Although wound care methods with current dressings have shown improved wound healing outcomes to some extent, they are still insufficient for refractory wounds (such as chronic wounds) or usually result in permanent scarring for deep wounds [7]. What’ s more, therapeutic effects of current wound dressings are challenged by flaws, such as non-adjustability features with wound environments, lacking high-tension endurance (such as for joints and necks), painful dressing-changing, etc [8]. Ongoing efforts have been made for advanced alternatives to regenerate skin with natural properties [9], of which smart wound dressings have drawn increasingly attention, exhibiting appealing potentials for wound management [6].

**Scheme 1:**
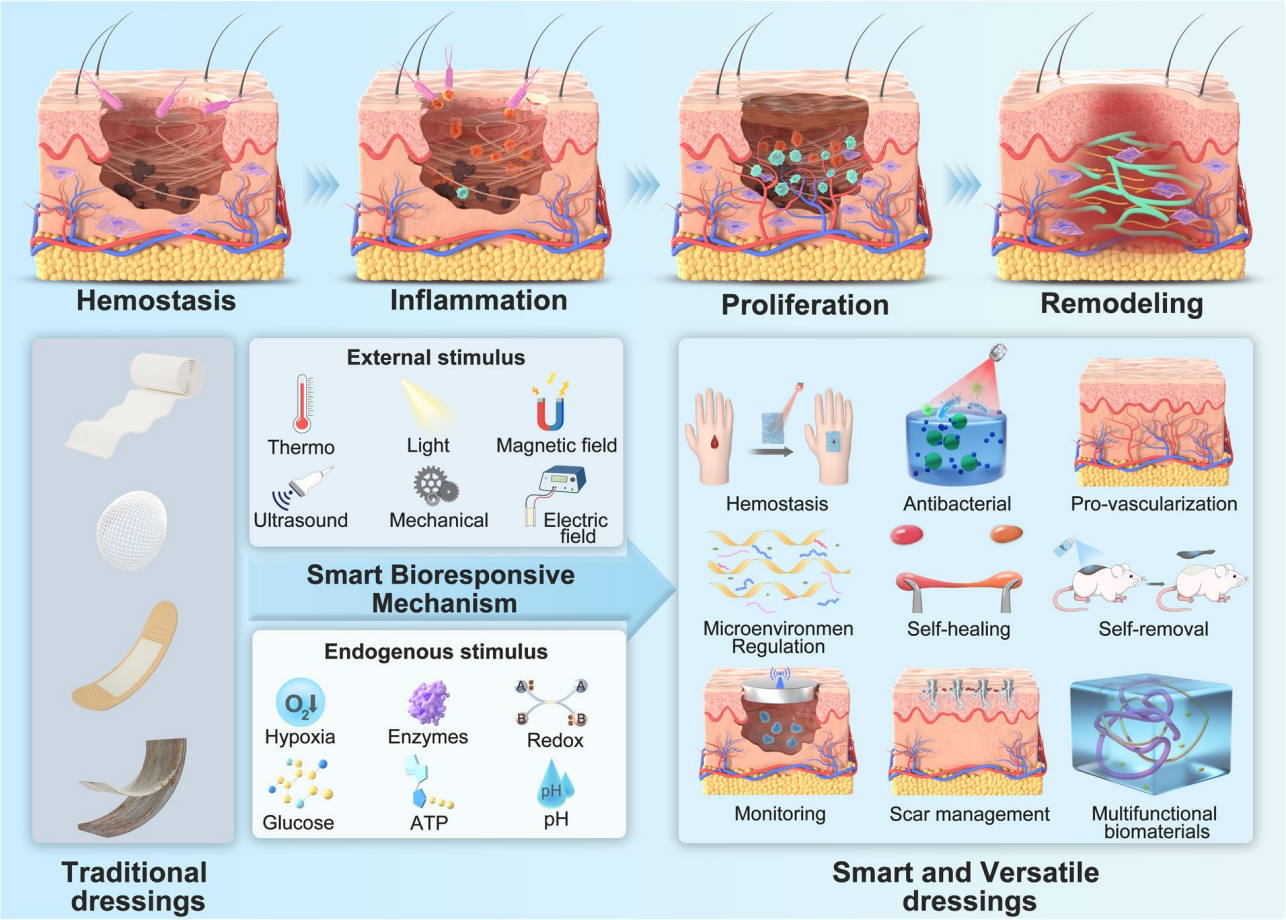
Schematic illustration of smart and versatile materials for cutaneous wound healing

Smart dressings for wound management have gradually evolved with the development of built-in sensors and intelligent materials that can respond to specific biological triggers (such as pH, temperature, enzymes, etc.) [8, 10]. Increasing understanding of healing processes and bioresponsive mechanisms contributes to various innovations in crucial fundamental researches. As a result, smart wound dressings are being developed for extensive applications, including active dressing, spatiotemporal drug delivery, regenerative template scaffold, real-time monitoring, specific in situ functionalities, etc [11]. Taking ‘drug delivery’ as an example, the treatment efficacy of therapies directly differs across administration methods (directly activated, progressively activated or self-regulated), necessitating the development of smart wound dressings to achieve precision of drug release [12]. To enable real-time monitoring, medical diagnosis based on bio-responsive materials must be enhanced towards non-invasive or minimally invasive approaches [13]. During wound healing process, diverse smart dressings may interact with the wound environment to identify the changes, facilitate hemostasis, regulate inflammation, control infection, inhibit the formation of chronic process and eventually foster wound healing [14]. To promote the advance of smart materials-based wound dressing, we herein presented a comprehensive and up-to-date overview of urgently needed wound dressings from general towards smart. This review covers wound healing, overview of biomaterials (e.g., original materials, categories by the form, operating strategies, etc.), responsive mechanisms (endogenous and exogenous stimulus), functional applications of cutaneous biomaterials, challenges (currently impeding clinical translation) and future perspectives (Scheme 1). The rationales for the design of smart wound dressing were particularly emphasized.

## An overview of wound healing

**Fig. 1:**
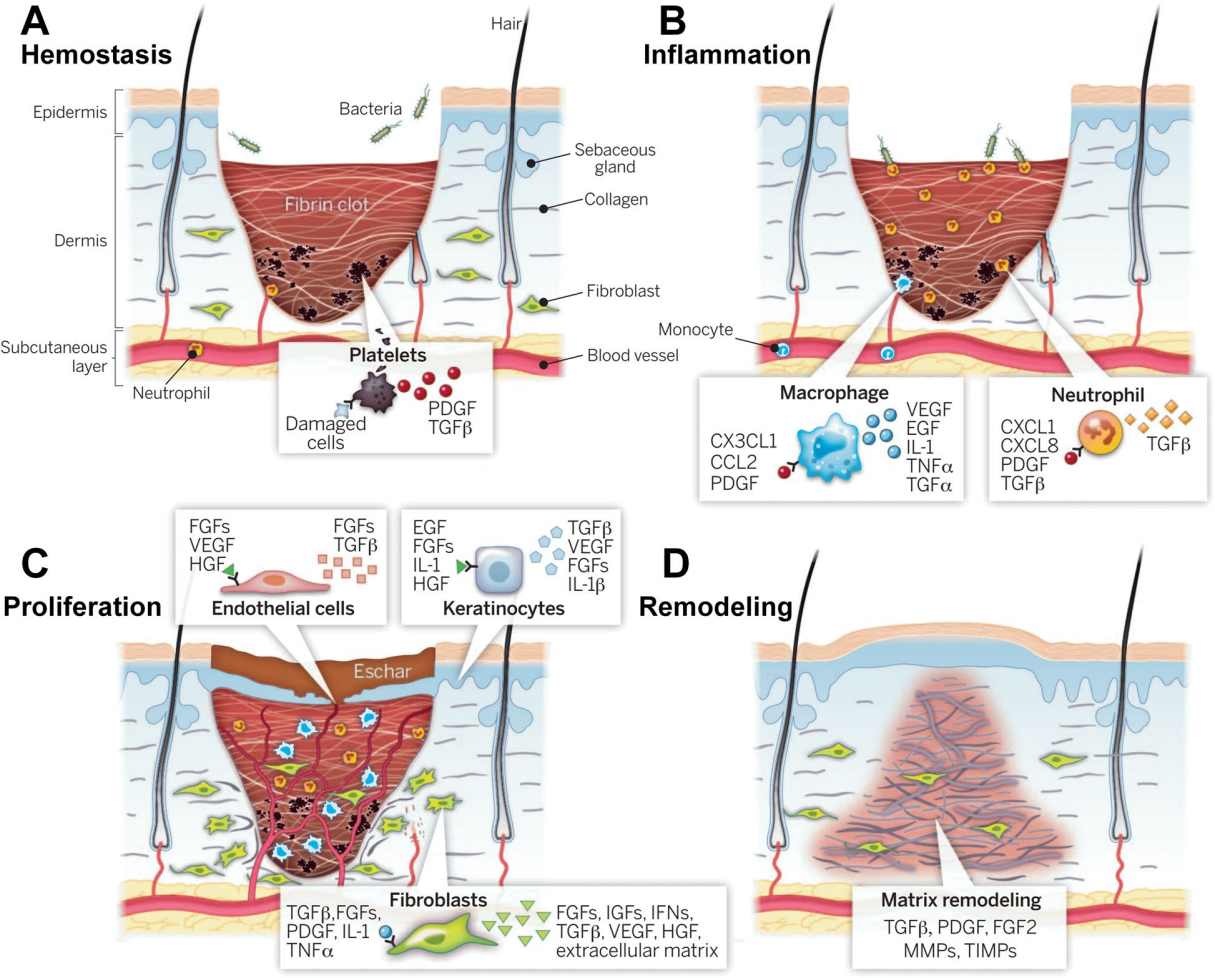
Schematic overview of healing processes. **A** hemostasis and coagulation. **B** inflammation. **C** proliferation. **D** remodeling. Reprinted with permission from ref [17]. Copyright 2014 AAAS

Wound healing is a complex physiological process governed by chemokines, cytokines, growth factors and several cell types [15]. Complex cellular and molecular responses are initiated rapidly after injury to restore skin barrier function and homeostasis, reducing infection and underlying complications [16]. Generally, physiological wound healing occurs in four stages [17], namely hemostasis, inflammation, proliferation and remodeling, which are all sequential, overlapping and precisely regulated (Fig. 1). Wound healing after skin injury is usually very efficient, but it may be compromised due to pathological alterations in the physiologically regulated biologic processes of normal healing. In general, pathological wound healing causes excessive healing (i.e., scar formation) or insufficient healing (such as chronic wounds) [18].

Although not completely understood, the pathophysiology of insufficient healing is well known for stagnating the inflammatory phase, rather than progressing to the healing process. Impaired vascularization with subsequent hypoxia, protracted and worsening inflammation, and immune cells’ failure in suppressing the infection of bacterial are all key problems impeding normal healing. Severe hypoxia causes the formation of massive non-viable/avascular tissues, providing an ideal environment for bacteria to multiply, with biofilm aggravating inflammation and inhibiting ECM deposition [19]. Excessive and prolonged expression of various inflammatory cytokines blocks the healing process from progressing to the proliferative stage. Microvascular insufficiency can also cause chronic hard-to-heal wounds, which are particularly frequent among patients with diabetic ulcers, vascular ulcers or pressure ulcers [11, 19].

Instead of regenerative restoration, most chronic wounds heal through fibrosis, which produces a large amount of connective tissue. Improper growth factor activity regulation leads to undesired fibroblast proliferation and neovascularization, as well as excessive synthesis of collagen and fibronectin [20]. Furthermore, protracted and undue wound contraction can exacerbate fibrotic scar formation, which is primary distinction between healed and physiological tissues [21]. The scar tissue matrix, typified by granulation tissue, is the final product rich in fibroblasts, capillaries, macrophages, granulocytes and collagens [11]. HTS and keloid, both featuring hyperproliferating fibroblasts and over-synthesized ECM, are two types of pathological scarring after injury [22]. Many growth factors that govern cellular proliferation and ECM synthesis, especially transforming growth factor-βs (TGF-βs), have a profound effect on scar’s progression and regression [23]. Besides, several types of cells, such as myofibroblasts and M2-like macrophages, are involved in extensive scarring. In HTS or keloids, myofibroblasts are less prone to apoptosis, with continued proliferate and remodeled collagen fibers [24].

## Existing biomaterials for wound healing

### Various polymers and form-based classifications of biomaterials for wound healing

Polymers of wound healing materials can be divided into three categories: natural, synthetic or hybrid polymers [25]. Natural polymers are biocompatible & biodegradable since they are derived nature, including proteins (e.g., gelatin, collagen, fibrin, etc.), polysaccharides [such as alginate, chitosan, hyaluronic acid (HA), etc.], etc [26]. Synthetic polymers with infinite diversity, are often more adaptable to fabrication processes and sterilization than their naturally occurring equivalents, such as Poly(lactic-co-glycolic acid) (PLGA), Poly(vinyl alcohol) (PVA), etc [27]. Hybrid polymers attempts to integrate the benefits of natural plus synthetic polymers via physicochemical links, with the goal of healing cutaneous wounds.

Over the past decades, biomaterials for wound healing have been developed for many forms and can be classified into scaffolds (fibrous, particulate, porous and printed scaffolds [28]), hydrogel [29], microneedles [30], microspheres [31], nanomaterials [32], etc. For instance, fibrous scaffolds comprised of various biodegradable polymers possess desired capability for wound repair, and nanofibers tends to be more preferable biomaterials than microfibers because the nanoscale features drive cells toward normal in vivo shape [33]. That is, each type of biomaterial has its own features and is applied according to the specific needs of skin repair.

### Cutaneous biomaterials in drug delivery

#### Therapeutic molecules delivered

Therapeutics of various types have been developed and applied to recover the dysregulated metabolic and signaling processes in the wound environment, including growth factors/cytokines (e.g., VEGF, PDGF), peptides, living cells (like stem cells), anti-inflammatory drugs, antimicrobial agents (like antibiotics, Ag NPs), gene transfer vectors (such as cDNA, siRNA, miRNA), etc [34]. However, conventional materials are difficult to adapt to the complex wound environment to achieve targeted delivery.

**Fig. 2:**
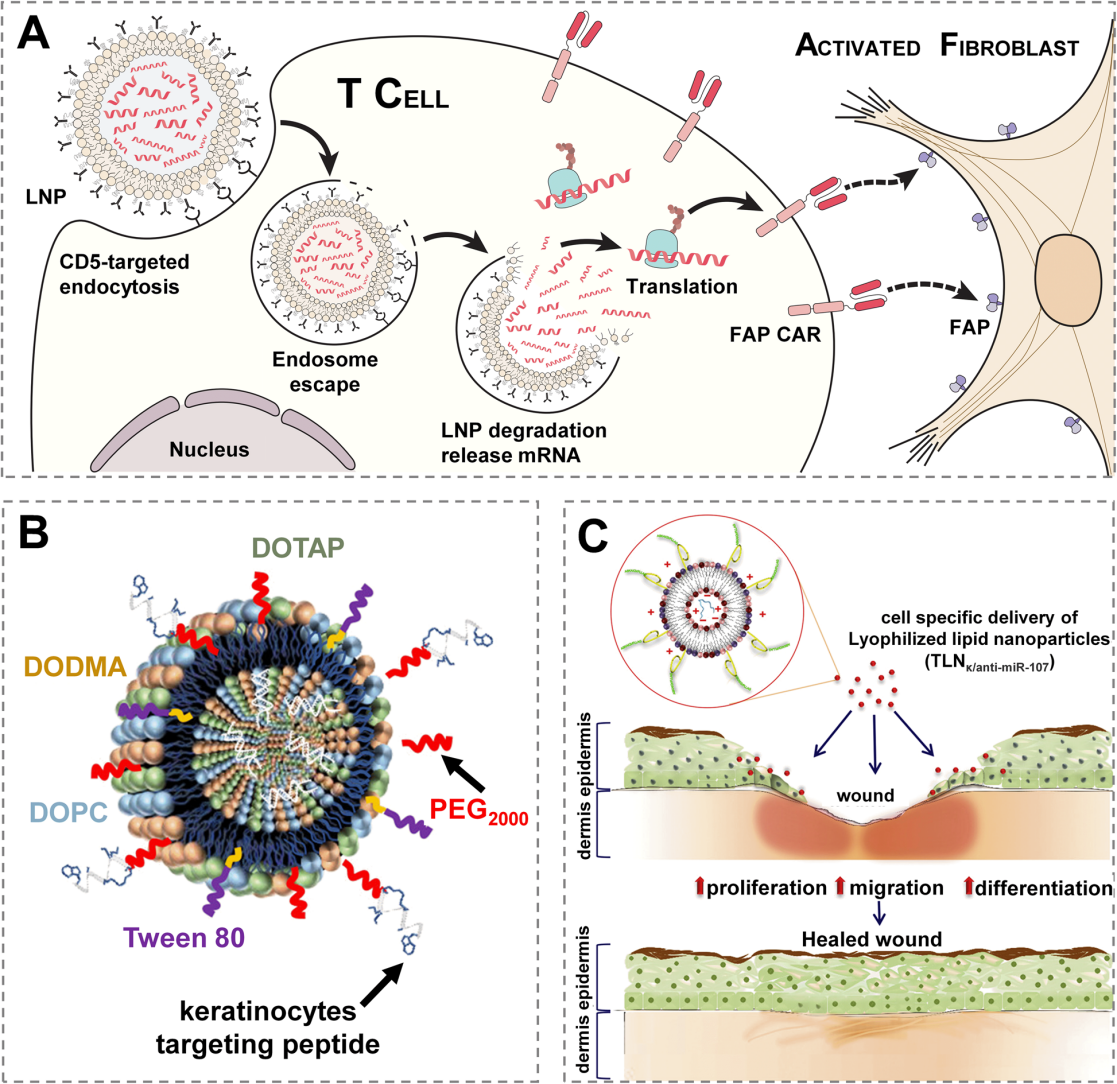
Typical applications of LNPs in controlled delivery of nucleic acids. **A** Schematic outlining the molecular process to create mRNA-based FAPCAR T cells in vitro with CD5-targeted LNPs. Reprinted with permission from ref [36]. Copyright 2022 AAAS. **B** Schematic representation of the keratinocyte-targeted LNPs (TLNPκ). Reprinted with permission from ref [38]. Copyright 2020 American Chemical Society. **C** Schematic representation of keratinocytes targeting LNPs (TLNκ) to deliver TLNκ/anti-miR-107. Reprinted with permission from ref [39]. Copyright 2018 Elsevier

Lipid nanoparticle [LNP] technology, which underpins the recent successes in COVID-19 vaccine development, is advantageous in delivery of nucleic acids (mRNA, siRNA, miRNA, etc.) [35]. By injecting CD5-targeted LNPs containing mRNA instructions to reprogram T lymphocytes, therapeutic chimeric antigen receptor (CAR) T cells entirely in vivo was firstly generated to reduce cardiac fibrosis (Fig. 2A) [36]. LNP also draws increasing attention in wound healing because of their significant functionalities, higher adherence to the skin and more effective diffusion through the skin barrier [37], such as keratinocyte-targeted lipid nanoparticles (TLNPκ) (Fig. 2B) [38] and locked nucleic acid (LNA) (TLNκ/anti-miR-107) for selectively deliver anti-miR-107 (Fig. 2C) [39].

#### Delivery mechanisms in wound healing

Systemic administration (via the circulatory system) and topical application are the two most common routes of drug intervention [22]. Systemic administration enables systemic perfusion of large doses of drugs, such as antibiotics for severe burns and persistent infections [40]. However, systemic administration can cause undesired tissue damage and toxic consequences due to the lack of targeting [41]. Instead, local delivery via topical application, painful transdermal delivery (e.g., intralesional injection [42]), and painless transdermal delivery (e.g., microneedles [43]) can markedly reduce the administration dose and thus minimize the reverse effects. Attributed to the vast exposed surface area of the lesion, topical administration is the most prevalent treatment strategy for cutaneous wound healing [44]. Bypassing enzymatic degradation in the gastrointestinal tract and first-pass metabolism in the liver, this approach brings biomolecules into direct contact with living blood vessels and cells in the vicinity of the wound, enhancing their efficacy [45]. Microneedles better facilitate drug transdermal delivery than other transdermal delivery systems, resulting in a more uniform application density with less pain.

### Biomaterials as templates for skin regeneration and wound healing

Traditional wound dressings have two main goals: to preserve wound beds and to create a favorable environment for wound healing. These products, on the other hand, are unable to replace destroyed tissue, such as a severely injured dermis [46]. Cellular tissue-engineered skin replacement solutions have been created to aid in the restoration of chronic ulcers that are difficult to heal. These substrates can be seeded with cells to create an engineered skin or implanted to help local cells recruit and proliferate. Current research is focusing on the usage of bioactive materials including collagen, HA, and chitosan. Collagen and HA, in particular, are biocompatible and biodegradable components of the ECM in live tissues that have demonstrated encouraging outcomes in vivo [47].

Wound healing is a dynamic and multi-staged process. Different wound healing stages have distinct molecular and physiological processes, resulting in different wound dressing requirements at various phases [48]. Wound dressings now available are often applied in a one-size-fits-all fashion, which is insufficient to fulfill the demands of wounds at various stages and are frequently not sensitive to changes in the environment. The following is a summary and discussion of recently emerged smart wound dressings.

## Engineering of smart wound dressing for cutaneous wound healing

**Fig. 3:**
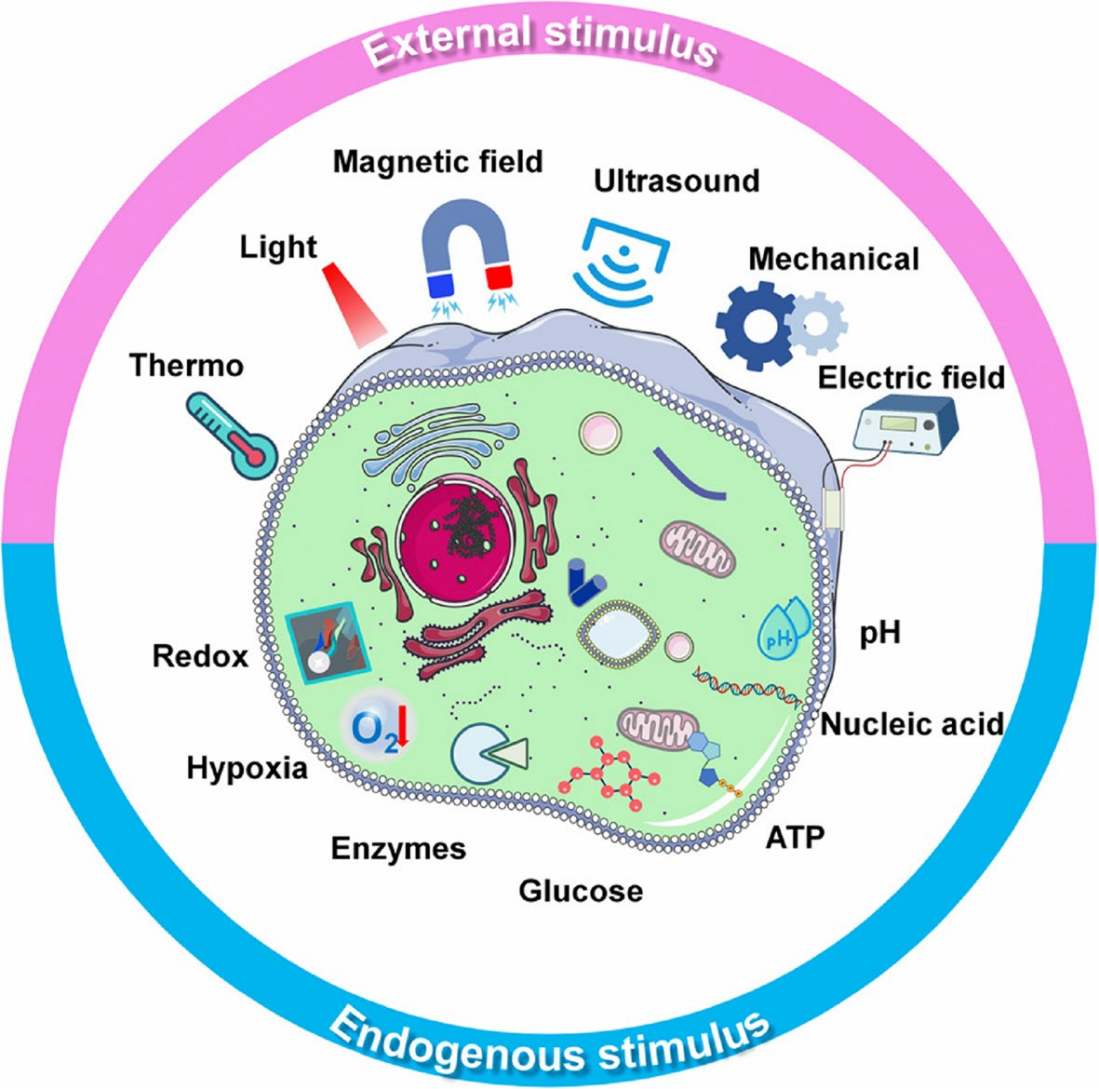
Endogenous and exogenous stimulus having been applied in the management of cutaneous wounds

Because of the exceptional design flexibility in installing various stimuli-sensitive motifs that respond to a wide range of endogenous or external inputs, smart biomaterials responding to the endogenous stimulus (like pH, redox, enzyme, hypoxia, glucose, etc.) or exogenous stimulus (including thermo, light, magnetic field, ultrasound, etc.) (Fig. 3 & Table 1) have been extensively exploited for cutaneous wound healing. The smart and versatile biomaterials can be cataloged into for hemostasis, antibacterial, anti-inflammatory, pro-vascularization, regulation of microenvironment, self-healing materials, self-removing materials, monitoring, scar management and versatile materials (Table 2).

**Table 1:** Key points and related notices of endogenous and exogenous stimulus

Stimulus	Key mechanisms or theoretical basis	Responsive components or devices	Notices or limitations	Ref.
**Endogenous stimulus**				
pH	① pH changes in disease: 4–6 for normal skin, decreased pH for acute wounds and increased pH for chronic wounds. ② Owing to ionizable groups’ protonation or the destruction of acid-cleavable bonds, pH-responsive materials experience physiochemical changes (including dissociation, swelling, degradation, shrinking, etc.).	Ionizable protonating copolymer or acid cleavable linkers.	① Little opportunity for chemical reaction. because of physiological pH fluctuations. ② Hard to enable ON/OFF switch resulted from continuous property,	[49, 50]
Redox	The redox potential of normal cells, normal tissues, and malignant tissues differs significantly, and concentration gradients of redox couples (such as glutathione/glutathione disulfide couple or diselenide linkage) can be applied to scavenge ROS generated in chronic wounds.	Diverse polymers such as iselenide-containing block copolymer.	Redox-responsive material may generate ROS on its own or be influenced by other unknown intracellular factors.	[51]
Enzyme	① High selectivity for substrate. ② Function under mild conditions. ③ Regulated in certain diseases. ④ The degradation of ECM (collagen, fibrin, elastin) is triggered by specific enzymes.	MMPs, protease, trypsin, furin, phospholipase, and galactosidase/glycosidase, etc.	The carriers’ resistance to enzyme assaults, as well as their stability in a diverse biological milieu, limit enzymatic triggers.	[52]
Hypoxia	Hypoxia is related to numerous diseases, including cancer, cardiomyopathy, ischaemia and chronic wounds.	Nitro aromatic derivatives, 2-nitroimidazole-grafted conjugated polymer, etc.	Hypoxia as a responsive trigger is still in its early stages of development, with limited reports on wound healing.	[53]
Glucose	① To achieve blood sugar monitoring and insulin injection (‘open-loop’ treatment). ② To design wounds dressings with glucose-responsive drug release or that consume the local glucose in situ.	Glucose oxidase (catalyzing the oxidation of glucose), yeast extract (converting glucose to ethanol)	Potential toxicity of ethanol produced by glucose consumption.	[54]
ATP	① The fluctuation of ATP concentrations between organelles, between external and intracellular environments, and between normal and diseased cells. ② The chemical energy of ATP is supplied to initiate self-assembling systems or it is utilized as a co-assembling component.	ATP-binding aptamers.	The selectivity of co-assembling systems is still an important issue.	[55]
**Exogenous stimulus**				
Thermo	① Temperature gradients and sensitivity that are abnormal in the tumor microenvironment or inflamed tissues are key physiochemical differences from healthy tissues. ② A phase transition occurs in thermosensitive polymers near their LCST values.	PNIPAM, synthetic polypeptides (including elastin-like polypeptides)	Challenges in clinical translation limited by non-degradability and toxicity.	[56]
Light	① Strong spatiotemporal resolution, full bioorthogonality, and precise wavelength and intensity tunability ② Light functions in the fabrication (such as initiating hydrogel gelation) and applications of biomaterials. ③ PDT (generating ROS) and PTT (generating heat) are two common applications of light therapy.	o-nitrobenzyl moiety, photosensitizers (PSs, for PDT), noble metal nanomaterials (for PTT), etc.	① Classical PSs are restricted by poor water solubility, photobleaching, short absorption wavelengths and undesirable bacterial selectivity. ② Overcoming lower energy conversion, poor photothermal stability, and complicated synthesis processes, NIR-responsive inorganic nanomaterials are more appropriate in PTT.	[57, 58]
Magnetic field	① Functioning in controlled release or redosing of drugs, mechanical stimulus of cells, and scaffold assembly into required structures. ② MHT (generating heat) demonstrates the thermogenic effect of magnetic fields as a trigger.	Iron oxide particles.	Currently, wound repair and tissue regeneration are not as common.	[59, 60]
Ultrasound	① Ultrasound affects the degradability and drug release kinetics of various small molecules and proteins. ② Easily accessible, painless, non-invasive, safe and able to penetrate tissues.	Degradable scaffolds (polyanhydrides, PEGs esters, and polylactide) and microstructures responsive to ultrasound (like liposomes, microbubbles and micelles)	Rarely used in wound healing.	[50]
Mechano-stimuli	① Mechanical cues prevalently participate in several biomechanical processes. ② The design of mechano-triggered biomaterials is advanced based on interactions between network components and non-covalent interactions.	① Mechano-sensitive protein transducers (such as Piezo1/Piezo2). ② Wearable, tensile strain-triggered drug delivery devices.	Trigger threshold is needed to be balanced.	[61, 62]
Electric fields	Possibility to trigger both cellular responses and biomaterial simultaneously.	Electroactive polymers, such as polyaniline, polypyrrole, polythiophene, ethylene vinyl acetate, and polyethylene.	Potentially invasive insertion of electrodes as triggers.	[63]

**Table 2:** Typical applications of smart and versatile biomaterials

Functions	Stimulus	Key components	Biomaterials designed	Observations	Ref.
Hemostasis	Visible light	Eosin Y (as a PS), GelMA, hemocoagulase.	The adhesive incorporated snake venom hemagglutinase and Eosin Y into GelMA, which rapidly cross-linked to form a hydrogel under visible light irradiation.	Based on mouse rat tail dissection and rat liver incision models, the bioadhesive was demonstrated to reduce the clotting time from 5–6 min to about 45s, with an approximately 80% reduction in bleeding volume.	[64]
Antibacterial	Light (PDT, PTT)	Gold nanoclusters (Au NCs)	Using an in situ method of growth, gold nanoclusters modified zirconium-based porphyrin metal–organic frameworks (Au NCs@PCN) were constructed.	Under near-infrared (NIR) laser irradiation, Au NCs@PCN can be heated to 56.2 °C and generate ROS, showing an effective killing effect on bacteria.	[65]
Anti-inflammation	ROS.	Nanozymes.	Using a one-step method that is simple and effective to fabricate ultrasmall Cu5.4O NPs (Cu5.4O USNPs)	Cu5.4O USNPs as nanozymes possessed multiple enzyme-mimicking and broad-spectrum ROS scavenging ability, as well as cytoprotective effects against ROS-mediated damage.	[66]
Pro-vascularization	Light.	Collagen.	Laser irradiation of the hydrogel generates cavitation gas bubbles, which rearrange the collagen fibers, resulting in stable microchannels (diameters: 20–60 μm).	Such 3D channels can enable the formation of artificial microvasculature by culturing endothelial cells, as well as cell media perfusion.	[67]
Regulation of wound microenvironment	-	Chitosan, HA, collagen, etc.	A hydrogel-based burn dressing prepared by one-pot fabrication process.	In rabbits, this hydrogel substance greatly expedited the healing of deep II degree burn lesions, indicating that it has great potential for trauma repair.	[68]
Self-healing wound dressing for motional wound	Mechano-stimuli.	GelMA and tannic acid (TA).	A self-heal double-network hydrogel fabricated with GelMA and tannic acid.	GelMA-TA gel has potential use in skin wound closure, sutureless gastric surgery, and strain sensing.	[69]
Self-removal wound dressing	Redox	Dopamine, PEG.	An injectable dopamine-based adhesive hydrogel containing PVI.	The adhesive strength is rapidly reduced by spraying Zn2 + solution, which was attributed to the established metal ion complexing between Zn2 + and PVI.	[70]
Monitoring	Thermo and light (UV).	UV light-emitting diodes, temperature sensor, and UV-responsive hydrogel.	A smart flexible electronics-integrated wound dressing real-time monitoring and on-demand therapy of infected wounds.	The combined UV-responsive antibacterial hydrogel and UV-light were activated at the commencement of infection, allowing the loaded antibiotic to be released in-situ into the wound site.	[71]
Scar management	Light.	ALA, HAase and Met.	An anti-scar strategy based on ALA-mediated PDT, combined with HAase based dissolving microneedles and Met.	HAase significantly enhanced the transdermal delivery efficiency of ALA. And HAase (as a “spear”) combined with Met (as a “shield”) greatly strengthed the anti-scar outcomes of PDT.	[72]
Versatile biomaterials	pH and glucose.	Phenylboronic acid, benzaldehyde, chitosan.	A pH/glucose dual-responsive Met release hydrogel dressings with adhesive and self-healing capabilities contributed by dual-dynamic bonding.	Based on a rat type II diabetic foot model, the hydrogel was demonstrated to promote wound healing by reducing inflammation and enhancing angiogenesis.	[73]

### Hemostasis

**Fig. 4:**
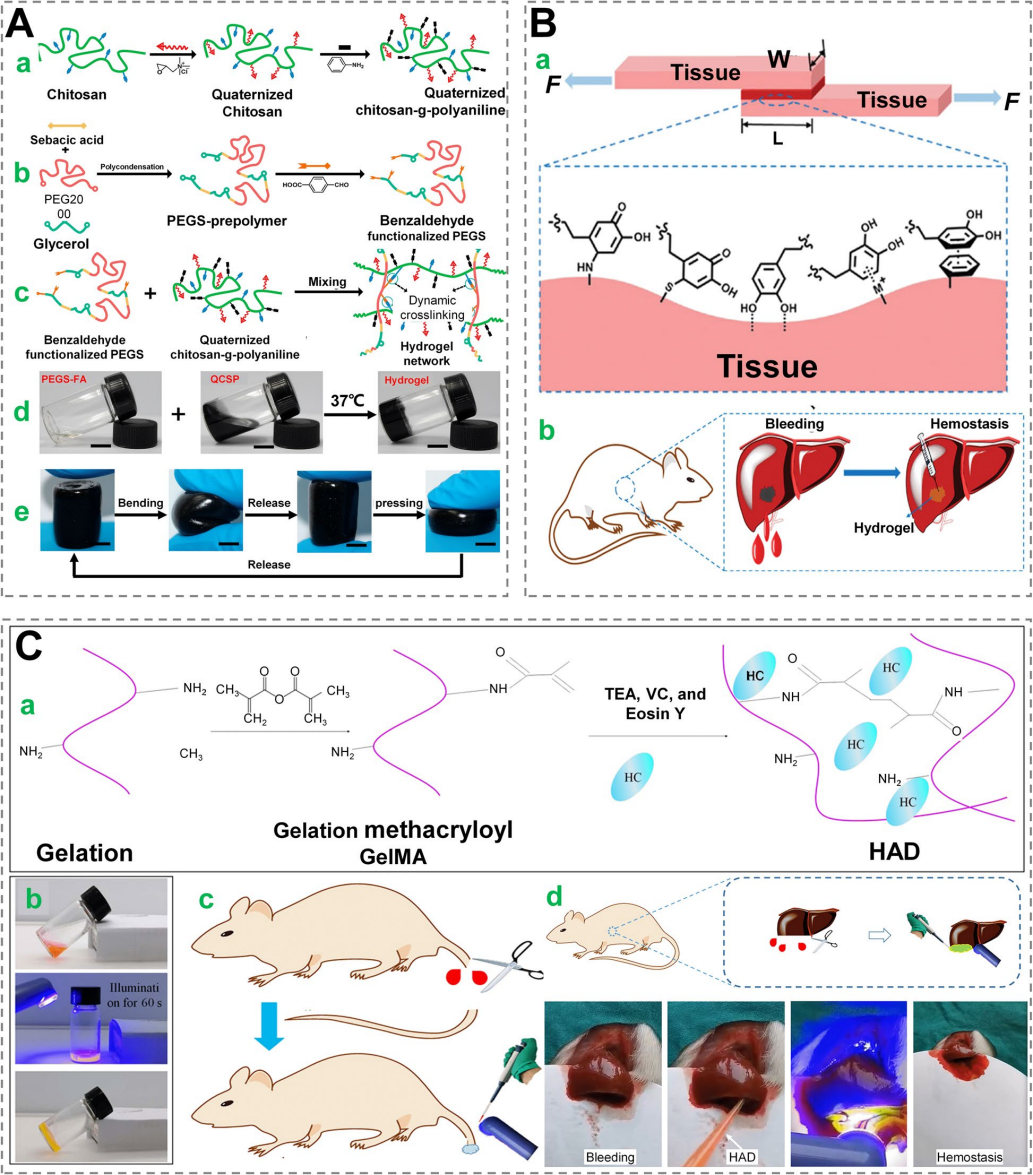
Wound dressings with hemostatic function. **A** Antibacterial, anti-oxidant, and electroactive dressing for wound repair based on quaternized chitosan-g-polyaniline (QCSP) and benzaldehyde group functionalized poly(ethylene glycol)-co-poly(glycerol sebacate) (PEGS-FA). Schematic illustration of this hydrogel’s synthesis. (a-c) Synthesis processes; (d) Photos of solutions and hydrogel (QCSP3/PEGS-FA1.5); (e) Two shapes (bending and pressing) of this hydrogel. Reprinted with permission from ref [77]. Copyright 2017 Elsevier. **B** Schematic diagram for the interface interactions between tissue and hydrogel, with desired properties such as hemostasis. Reprinted with permission from ref [88]. Copyright 2021 Elsevier. **C** A visible light-stimulating hemostatic adhesive (HAD). (a) Schematic overview of the visible light–responsive photopolymerization device; (b) Before (top), during (middle), and after visible light illumination, a digital image of the HAD gelling transition was taken (bottom); (c) Diagram depicting the formation of rat tail hemorrhage and a hemostatic model; (d) Schematic presentation of hemostatic processes and hemostatic assay using HAD based on a serious liver wound model. Reprinted with permission from ref [64]. Copyright 2021 AAAS

Hemostasis helps in the initiation of the inflammatory process and aids the following proliferation and remodeling stages, learning how to halt bleeding promptly and precisely is an important goal in the early treatment of skin wounds [22, 74]. In addition to physical methods such as bandaging and chemical ones like drugs, hemostatic materials such as chitosan, alginate, and fibrin-based materials are commonly used to halt bleeding in cutaneous wounds [75]. Hemostasis, being a fundamental function, is frequently employed in conjunction with additional functions. When combined with the adhesive properties of a mucoadhesive hydrogel, hemostasis can be achieved by closing the wound and the mucoadhesive hydrogel also adheres seamlessly to the wound site for a long time, reducing the potential infection resulted from the contact with the external environment [76]. For example, chitosan with a positive charge can improve adherence to cutaneous tissues via electrostatic contact (Fig. 4A) [77]. Besides, polymeric foams / sponges based on silicone, polydimethylsiloxane, polyurethane in combination with CO_2_, N_2_ or O_2_ as blowing agents may possess the effects of stopping bleeding and (or) promoting wound healing [78–81]. For example, incorporating phenolic acid into the network of polyurethane foam can obtain shape-memory performance, antibacterial and antioxidant properties, and ultimately reduce bleeding related deaths and subsequent infections [82].

Based on Schiff base reaction, the amino group on the tissue may cross-linke the aldehyde group, enabling enhanced tissue adherence [83, 84]. Inspired by the superb adhesion of marine mussels in wet environments, the dopamine-based theory of underwater wet adhesion has guided the development of many dopamine-based bio-tissue adhesive hydrogels [85, 86]. For instance, hydrogel patches with catechol/pyrogallol grafted hyaluronan were designed to serve as drug-loaded ready-to-use tissue tapes in aiding cutaneous wounds [87]. However, these materials with limited hemostatic effect lack anti-inflammatory and anti-infective properties, as well as precise and controllable applications to facilitate wound repair. By modulating hydrogel microstructure, Teng et al. fabricated two hydrogels with 16–18 μm pore size, which were demonstrated not only to achieve significantly faster hemostasis (~ 14 s) and lower blood loss (~ 6%) than fibrin gels, but also to facilitate wound repair (Fig. 4B) [88]. Furthermore, Guo et al. [64] developed a visible light-stimulating hemostatic bioadhesive that was inspired by snake venom hemagglutinase activity. The adhesive incorporated snake venom hemagglutinase and eosin into GelMA, which rapidly cross-linked to form a hydrogel under visible light irradiation to achieve rapid hemostasis and wound closure through mechanisms such as physical adhesion, induction of platelet aggregation and activation. Based on mouse rat tail dissection and rat liver incision models, the bioadhesive was demonstrated to reduce the clotting time from 5 to 6 min to about 45s, with an approximately 80% reduction in bleeding volume (Fig. 4C).

### Antibacterial

Bacterial infection during wound healing is an inevitable yet urgent problem that must be addressed. Antibacterial materials that are often employed include (but not limit to) antibiotics, metal ions (Ag^+^, Cu^2+^, etc.), antibacterial peptides, cationic polymers (quaternary chitosan), biomimetic nanozymes and graphene [89]; Whereas antibacterial strategies include chemodynamic therapy (CDT), PDT, PTT, MHT, etc [72, 90]. Wound dressings should be tailored to the diverse types and characteristics of wound in order to achieve the goal of effective healing. The sections that follow provide a brief overview of onesome of the most common antibacterial materials.

**Fig. 5:**
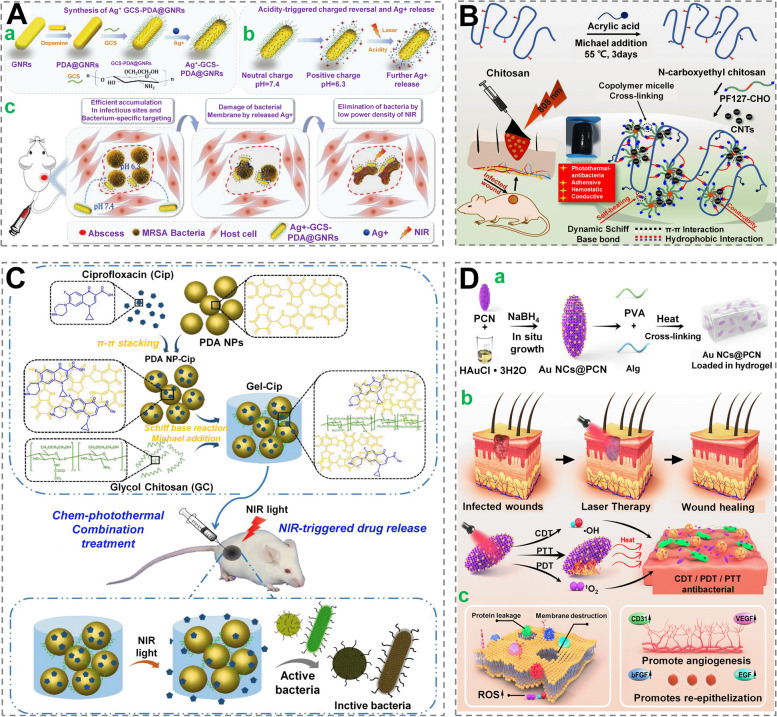
Wound dressings with antibacterial function. **A** PDA-coated gold nanorods (GNRs) were used to create a chemo-photothermal therapy platform. (a) Schematic of material synthesis; (b) Schematic of charge reversal and Ag + ion release as a result of acidity; (c) Schematic of bacterial-specific targeting and chemo-photothermal combo treatment. Reprinted with permission from ref [94]. Copyright 2018 Springer Nature. **B** Schematic representation of a self-healing hydrogel dressing which was conductive and adhesive using Pluronic F127/carbon nanotubes (PF127/CNT) and N-carboxyethyl chitosan (CEC). Reprinted with permission from ref [102]. Copyright 2020 Elsevier. **C** The drug reservoir was made by mixing ciprofloxacin (Cip, a strong antibiotic)-loaded PDA NPs and glycol chitosan (GC) to generate an injectable hydrogel (PDA NP-Cip/GC hydrogel, dubbed Gel-Cip). Reprinted with permission from ref [103]. Copyright 2018 Elsevier. **D** Gold nanoclusters modified zirconium-based porphyrin metal–organic frameworks (Au NCs@PCN) were designed for Infected Diabetic Wound Healing. (a) Diagram of the Au NCs@PCN fabrication method; (b) High-temperature death of multidrug resistance bacteria under NIR radiation by CDT, PDT, and PTT; (c) Bactericidal via altering bacterial membrane structure and encouraging angiogenesis and epithelial cell healing by upregulating the expression of associated factors. Reprinted with permission from ref [65]. Copyright 2022 American Chemical Society

Antibiotics have long been the preferred treatment for infected wound, where they are utilized alone or in combination with biomaterials to fabricate antimicrobial wound dressings. Inorganic metal NPs are being extensively investigated for antibacterial purposes, while concerns about metal biotoxicity and long-term retention have yet to be resolved [91, 92]. With the advent of nanotechnology, Ag^+^, an ancient antibacterial agent with excellent broad-spectrum antimicrobial properties, has also been employed to form new Ag-containing antibacterial materials in wound repair such as Ag NPs generated in situ on the surface of a porous thermoplastic polyurethane film [93]. Based on the understanding that the toxicity of Ag ions is concentration-dependent, Liu et al. [94] designed a new functional material loaded with a minimum effective concentration (1 µg/mL) of Ag ions onto the surface of Au nanorods modified with ethylene glycol chitosan, which can target the surface of bacteria and release Ag ions in situ to kill them. Besides, in a mouse subcutaneous abscess model, Au nanorods showed synergistic antibacterial and PTT actions in encouraging wound healing (Fig. 5A).

Antimicrobial peptides are natural immune defense substances with broad-spectrum antibacterial activity and biocompatibility. It is widely assumed that their bactericidal mechanism involves electrostatic targeting of bacteria’s surface, which leads to bacterial death by disrupting the cell membrane. Notably, antimicrobial peptides exhibit biological functions such as enhancing immunity and promoting wound healing, and hence offer a wide range of applications in wound repair [95].

Antibiotics resistance, biological toxicity, metal antimicrobials’ long-term retention and other limitations have prompted researchers to look into novel antimicrobial strategies such as cationic compounds, phototherapies, biomimetic nanozymes, etc. [75] Antibacterial action has been demonstrated for cationic chemicals for the positive charges, which tempt negatively charged bacteria and then kill them by disrupting their cell membrane [96]. Chitosan has been utilized to interact with konjac glucomannan, gelatin, PVP/agar, carbon dots, lignin/PVA, etc., with or without the application of responsive stimulations [97–99]. Grafting cations [like quaternary ammonium and poly(aminoethyl)] onto chitosan’s main chain might also boost its antibacterial action [100, 101]. Liang et al. created a versatile nanocomposite hydrogel consisting of N-carboxyethyl chitosan and other components (Pluronic F-127 plus carbon nanotubes), which was demonstrated to obtain significant prospective as a PTT for infected wounds (Fig. 5B). [102].

Furthermore, Gao et al. also devised a system with photothermal effects, possessing a controllable antibiotic release initiated by NIR light (Fig. 5C) [103]. Nanozymes are artificial enzymes that possess the benefits of high stability, simplicity of preparation & manufacture and low cost over natural ones [104]. The antibacterial activities of nanozymes are mediated by three primary mechanisms: extracellular DNA clearance, ROS control and HOBr/Cl formation. Exogenous ROS has universally been reported to kill bacteria irreversibly and to prevent the bacterial biofilms formation. Inspired by this, nanozymes for ROS production are mostly designed by mimicking natural peroxidase and oxidase, which can generate H_2_O_2_ and highly active ·O_2_^−/1^O_2_ by catalyzing oxygen, both of which possess good antibacterial effects (Fig. 5D) [65].

### Anti-inflammation

The second (inflammatory) phase of wound healing concentrates on bacterial elimination and debris clearance. Since controlling inflammation is so crucial for wound repair, it should be a top priority when designing wound dressings [105]. Exaggerated inflammation may result in significant oxidative stress with a surge of ROS [106], impeding the healing of cutaneous wounds, such as chronic wounds caused by diabetes and vascular disease, which are frequently associated with persistent excessive inflammation [107]. In addition, excessive inflammation has been linked to scar formation during wound healing [108]. Antioxidants are substances capable of trapping and neutralizing free radicals in order to eliminate their harmful effects on the body. Examples of antioxidants include polyphenol antioxidants (curcumin, tea polyphenols, resveratrol, anthocyanins and some flavonoids [109–113]), molecules with catechol structure (like dopamine [114]), endogenous antioxidants (like superoxide dismutase, SOD [115]), etc.

**Fig. 6:**
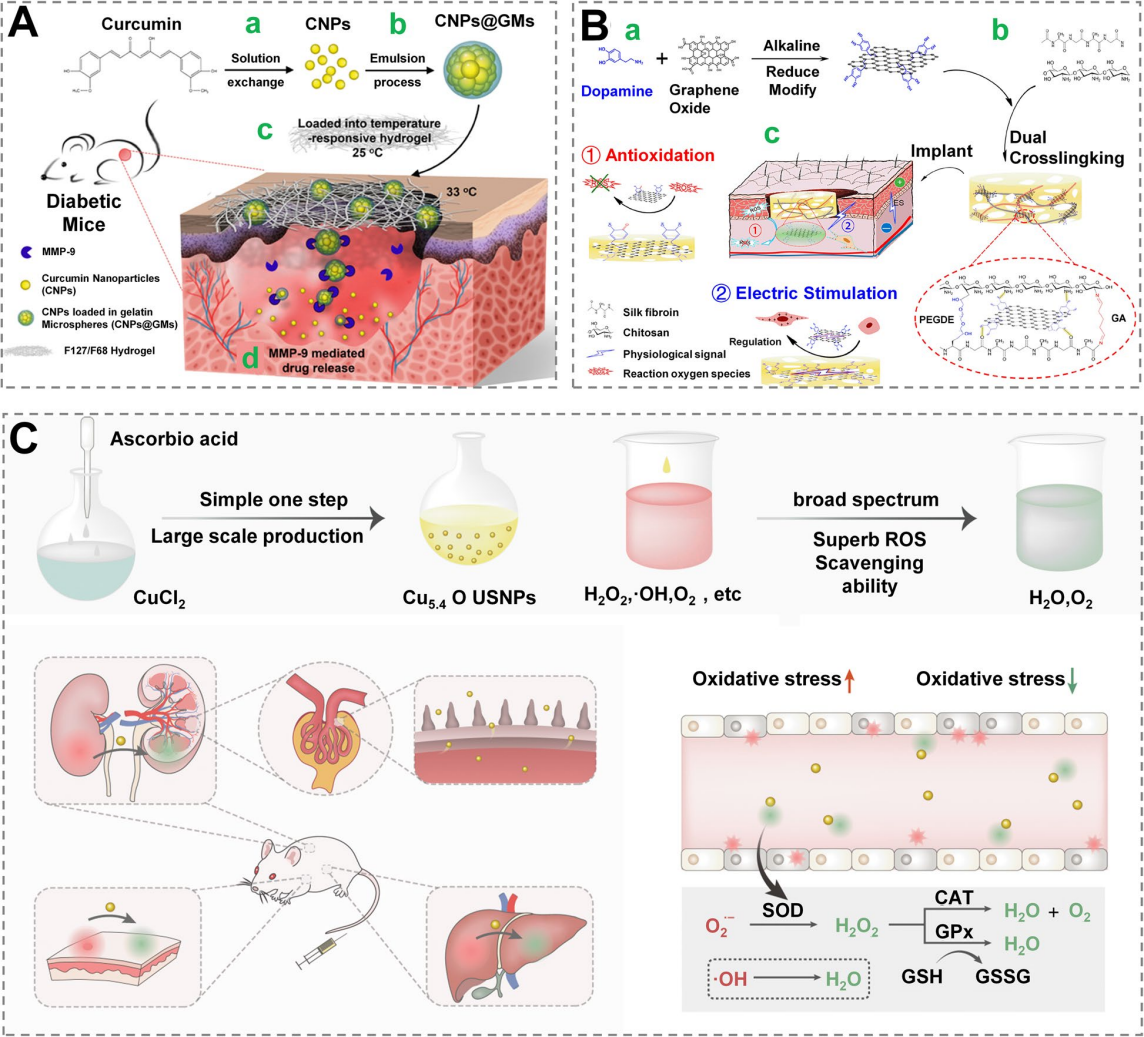
Wound dressings with anti-inflammation function. **A** Curcumin was delivered via a thermosensitive hydrogel containing the nanodrug in the form of gelatin microspheres (GMs) (Cur) to improves diabetic wound healing. (a) Solution exchange method for producing pure CNPs. (b) The emulsion process loads CNPs into GMs, resulting in CNPs@GMs. (c) In diabetic mice, CNPs@GMs were combined with a thermos-sensitive hydrogel and applied to the wound. (d) GMs were destroyed by MMPs in the microenvironment of a nonhealing wound, and the medication was precisely released. Reprinted with permission from ref [117]. Copyright 2018 American Chemical Society. **B** Electroactive and antioxidative scaffold was fabricated for wound healing. (a) PDA’s synthetic approach decreased and functionalized pGO. (b) The pGO-CS/SF scaffold is formed with dual cross-links. (c) The skin wound defect was repaired using the scaffold. During wound healing, the scaffold exhibits antioxidative properties and electrical stimulation of the skin tissue. Reprinted with permission from ref [118]. Copyright 2019 American Chemical Society. **C** Using an efficient and simple one-step technique, ultrasmall Cu5.4O NPs (Cu5.4O USNPs) were created as nanozymes with numerous enzyme-mimicking and widening ROS scavenging capacity against broad ROS-related illnesses, including acute kidney injury, acute liver injury and diabetic wound healing. Reprinted with permission from ref [66]. Copyright 2019 American Chemical Society

Systemic delivery of curcumin is limited by its poor stability and water insolubility, while possessing anti-inflammatory activity effectiveness equivalent to steroid or nonsteroidal agents. To address this issue, Wathoni et al. constructed a composite hydrogel containing curcumin and 2-hydroxypropyl-γ-cyclodextrin, and it was proved to greatly improve the disadvantages of curcumin itself [116]. When curcumin NPs were loaded into gelatin microspheres, the newly constructed system was not only biocompatible but also effective in enhancing diabetic ulcer healing(Fig. 6A) [117].

Considering that dopamine molecules possess antioxidant activity (because of the catechol structures), Tang et al. also fabricated a hydrogel (based on dopamine) with antioxidant effects, whose beneficial effects on wound healing were verified (Fig. 6B) [118]. Zhang et al. created a bandage containing SOD that enhances chronic wound healing by catalyzing the breakdown of superoxide radicals to H_2_O_2_, which is subsequently converted to water and oxygen, thereby eliminating excess ROS [115]. Recently, Liu et al. developed a simple, green and large-scale fabrication of ultra-small Cu5.4O nanozyme, which has a variety of enzyme-mimicking properties, broad-spectrum and high-efficiency ROS scavenging ability, and can be used to repair chronic wounds such as diabetic foot ulcer (Fig. 6C) [66]. As a result, nanozymes have the capacity not only to control ROS for destroying undesirable components (such as bacteria), but also to alleviate inflammation (e.g., those associated with bacterial infections), as previously reported [119]. Therefore, it is necessary to optimize the application mode of traditional antioxidants to regulate wound inflammation. Furthermore, emerging strategies such as nanozymes will also be a nonnegligible research direction.

### Pro-vascularization

Angiogenesis is crucial for wound healing, especially during the proliferation stage. Many efforts have been made to improve this process through the use of proteins (such as growth factors), small molecules/drug-like compounds, gene/nucleic acid delivery, and cell therapies [120]. As one of the most important pro-vascularization factors, VEGF can be encapsulated into PLGA microspheres and PLGA NPs in scaffolds (based on chitosan/HA & fibrin), which possess sustained release of VEGF, positively inducing angiogenesis in diabetic mice with chronic infected wounds [121]. Many other proteins, such as the hormone erythropoietin (EPO), peptides, insulin, blood derived factors, stromal-cell derived factor-1 (SDF-1), can also accelerate angiogenesis in wound healing models [122, 123]. EPO has been utilized to treat ischemic injuries when significant revascularization is necessary to restore sufficient blood flow, and it has been claimed that EPO acts in tandem with VEGF to enhance wound angiogenesis [124].

**Fig. 7:**
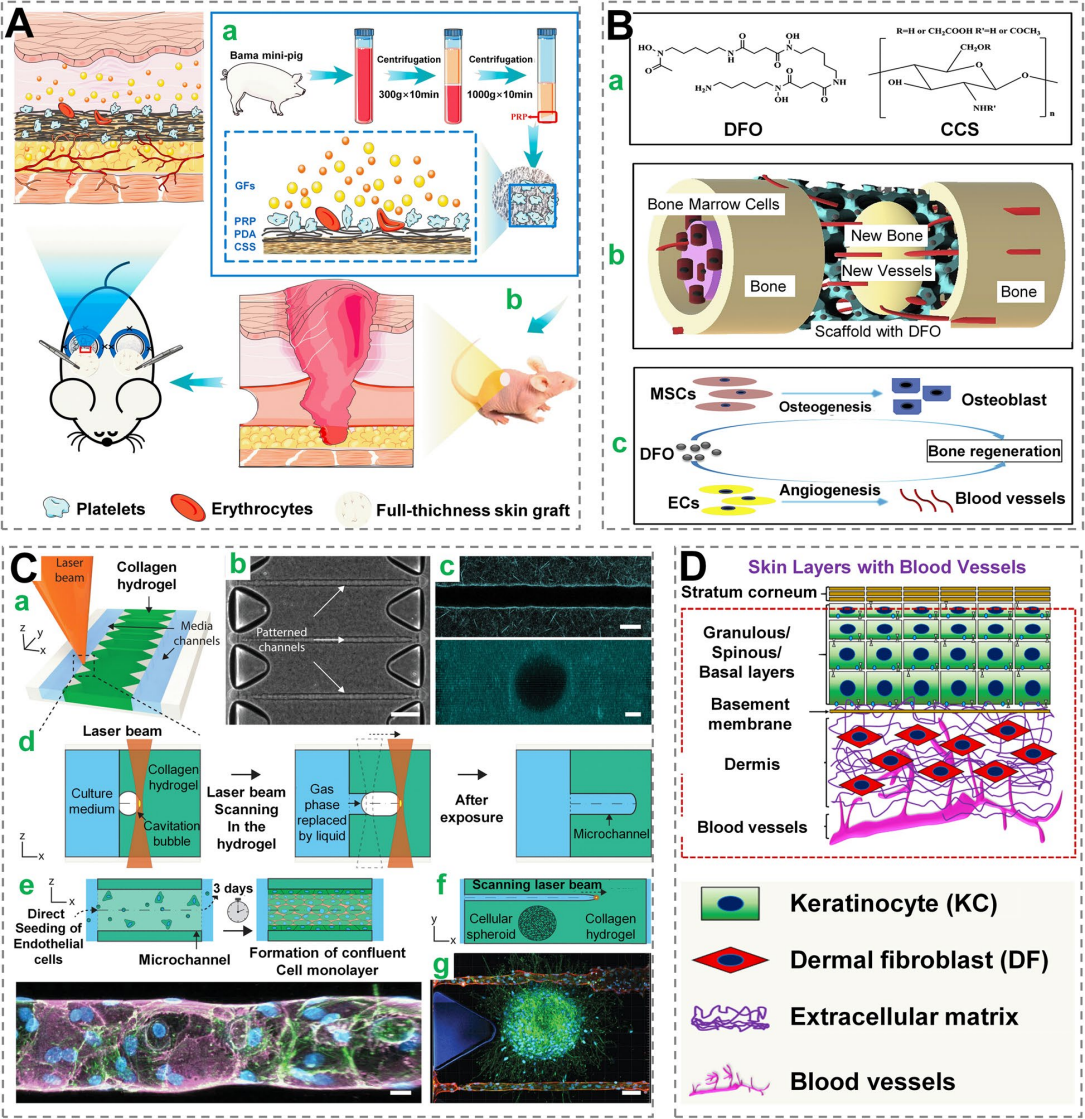
Wound dressings with pro-vascularization function. **A** PDA modified collagen sponge scaffold (pDA-CSS) was fabricated to deliver PRP for skin repair. (a) PRP is coupled with pDA-CSS to promote the release of growth factors (GFs). (b) Attributed to pro-vascularization and pro-proliferation, the pDA-CSS delivering PRP achieved one-step to accelerate wound healing. Reprinted with permission from ref [125]. Copyright 2021 Elsevier. **B** A biodegradable scaffold was fabricated to release DFO for angiogenesis and osteogenesis. (a) DFO and charged carboxymethyl chitosan have similar chemical molecular structures (CCS). (b) Illustration angiogenesis benefiting from scaffold. (c) DFO promoted bone repair in MSCs and vascular endothelia cells (ECs) via a biological mechanism. Reprinted with permission from ref [130]. Copyright 2018 Elsevier. **C** Cavitation molding with a 3D laser for vascularized tissue models. (a) Schematic of femtosecond laser exposure in situ patterning of collagen hydrogel. (b-c) Designed microchannels in a collagen hydrogel, brightfield and confocal pictures (d) Diagram of the dynamics during laser-induced cavitation and after the bubbles have collapsed and the matrix has relaxed. (e) Endothelial cells are seeded directly into the designed channels, resulting in the development of an artificial blood artery. (f-g) Developed vascularized glioblastoma spheroid model with encapsulated U87 glioblastoma cellular spheroids, with concept sketch and confocal fluorescence photographs. Reprinted with permission from ref [67]. Copyright 2022 Wiley-VCH. (**D**) Endothelial cell networks, dermal fibroblasts, and multilayered keratinocytes were used to create a schematic depicting the arrangement of skin layers. Reprinted with permission from ref [136]. Copyright 2021 IOP Science

Platelet-rich plasma (PRP), which contains quantities of growth factors is generated via gradient centrifugation of whole blood and can induce neovascularization to foster wound healing (Fig. 7A**)** [125]. As a non-coding RNA, miRNA is around 21–25 nucleotides long and plays an important role in the modulation of gene expression by binding to targeted mRNA, exerting an inhibitory effect on mRNA synthesis or inducing effect on mRNA degradation [126]. Several miRNAs (miR-21, miR-23a, etc.) have been reported to regulate angiogenesis via scaffolds and hydrogels [120]. What’s more, small molecules/drug-like compounds [e.g., NO, ATP, statins, deferoxamine (DFO), etc.] promote angiogenesis and aid in the recovery of defective wound healing [127, 128].

In hypoxia-induced wounds, hypoxia-inducible factor-1 (HIF-1) governs cell sensitivity to variations in oxygen levels and plays a pro-vascular role [129]. DFO is an iron chelator that has been used to stimulate HIF-1 accumulation in cells by simulating oxygen deprivation. Yan et al. created a biodegradable scaffold that uses surface aminolysis and a layer-by-layer construction approach to regulate DFO release, which is important for angiogenesis, as well as osteogenesis (Fig. 7B) [130].

Recent advances in 3D printing have eased the fabrication of vascular scaffolds, opening up new avenues for tissue vascularization [131]. In the last decade, substantial progresses have been achieved in developing synthetic biomaterials and printing processes, greatly enabling the bio-printing constructions of indirect patterning vascular networks [132]. Nevertheless, artificial vessels via traditional strategies are often > 100 μm in diameter, limiting their applications [133].

As laser photoablation technology advances, vascular networks can be generated with increased resolution and complexity, the photothermal process, however, may compromise hydrogel integrity and cell survival. To address these issues, Alessandro Enrico et al. employed femtosecond laser irradiation to create tunnels and cavities in collagen-based hydrogels, whose diameters varied from 20 to 60 μm (Fig. 7C). In this process, laser irradiation on this hydrogel triggered the formation of cavitation bubbles, which reconstructed the collagen-based fibers, contributing to durable microchannels [67].

Different in-vitro skin models have been manufactured and marketed since Bell et al. initially reported human skin-like structures in the early 1980s [134]. Balatazar et al., for example, used 3D bioprinting to create vascularized skin replacements that were as complex as natural skin tissues [135]. Natan R Barros et al. developed a 3D skin model comprising multi-layer keratinocytes, fibroblasts and endothelial cell networks (Fig. 7D). Mechanical characteristics of GelMA-based bioinks combined alginate (with various portions) indicated that a mixture of 2% alginate and 7.5% GelMA may be properly modeled to enhance the viability of endothelial cells in bio-printing endothelium [136]. Although researchers aimed to create more integrated structures near the natural niche, the intricacy of each skin layer still has to be enhanced to develop an optimum vascularized skin model.

### Regulation of wound microenvironment

Physical factors such as temperature, humidity, electric, and magnetism in the traumatic microenvironment all have varying degrees of influence on wound healing. Xu et al. [137] designed a bilayer silicone material with different pore sizes that has excellent porosity and water vapor transmission rate and can maintain the moist microenvironment of traumatic surfaces. The results showed that applying this silicone material to a mouse model of total skin defect could effectively promote wound re-epithelialization and vascularization, hence accelerating wound healing [137]. The ECM plays a fundamental role in cellular proliferation, migration and differentiation, as well as other biological activities in the traumatic microenvironment, and functional materials that mimic the ECM structure can enhance cellular bioactivity and subsequently promote wound healing. The electrostatic spinning technique is a recently emerging method for the synthesis of materials that can produce functional materials with a structure comparable to natural ECM, possessing large-scale surface and high-porosity, which can facilitate wound repair by promoting cellular proliferation and migration [138].

**Fig. 8:**
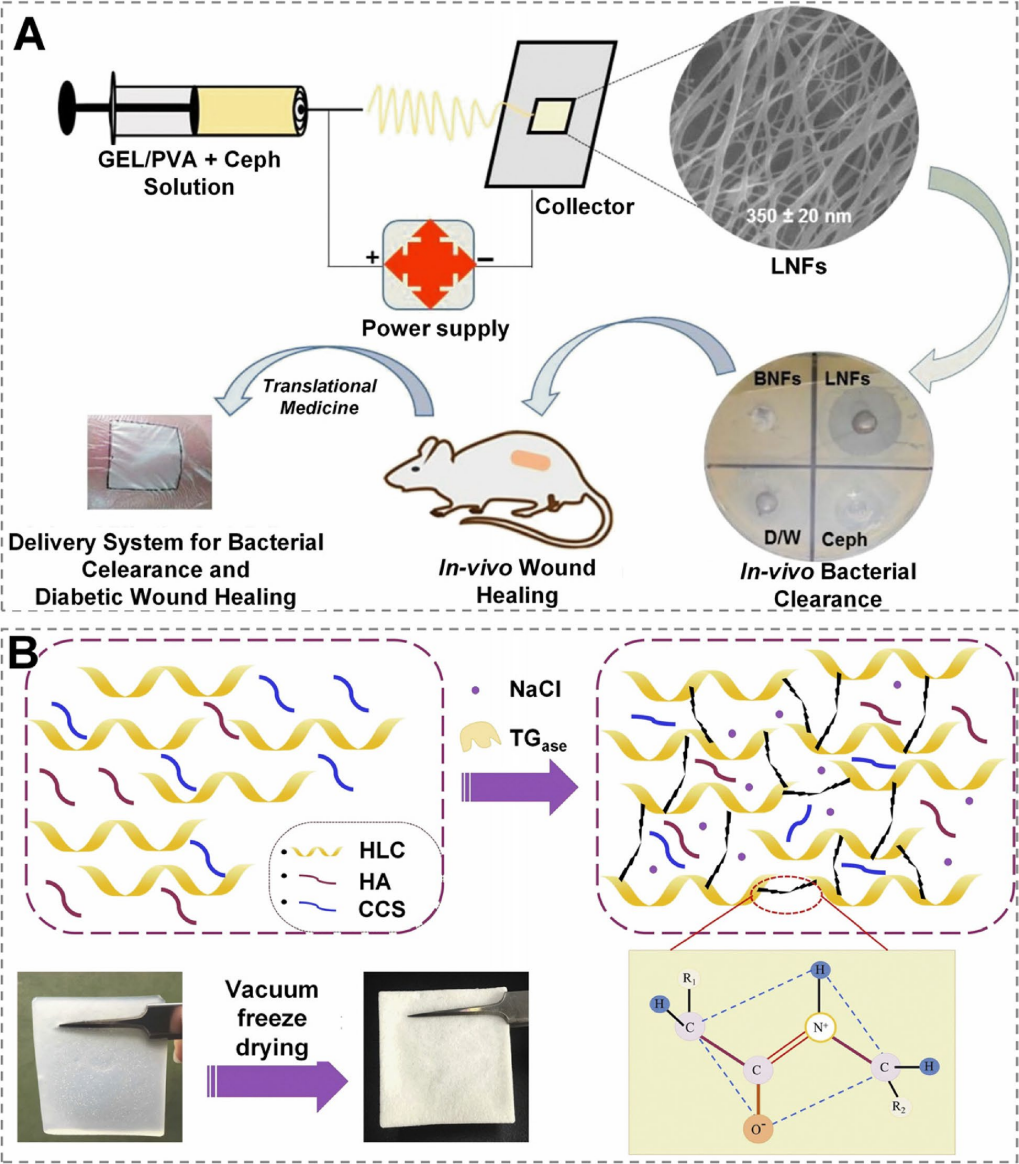
Wound dressings with wound microenvironment regulation function. **A** Schematic displaying gelatin (GEL)/polyvinyl alcohol (PVA) electrospun nanofibers (BNFs), which was synthesized using electrostatic spinning to load cefradine as a bionic antibacterial functional material. Reprinted with permission from ref [141]. Copyright 2021 MDPI. **B** HA, carboxylated chitosan (CCS) and human-like collagen (HLC) were mixed to ECM, and glutamine transaminase (TG) was utilized as a crosslinker in a schematic one-pot production procedure of hydrogel-based burn dressing. Reprinted with permission from ref [68]. Copyright 2020 Elsevier

To develop a bionic antibacterial functional material, Razzaq et al. [139] used electrostatic spinning to synthesize gelatin/polyvinyl alcohol fibre films loaded with cefradine (Fig. 8A). This study showed that the material not only showed good antimicrobial properties, but it also effectively promoted wound healing when applied to diabetic mouse wounds. Hydrogel materials are also favored due to their natural three-dimensional structure, and Lei et al. [68] used glutamine aminotransferase as a cross-linking agent to synthesize a collagen/HA/carboxylated chitosan hydrogel mimicking natural ECM, with a characteristic three-dimensional porous structure (pore size: 90.43 ± 5.57 μm) and good mechanical properties [modulus of elasticity of ( 480.43 ± 15.82) kPa and a tensile strain of 55.23% ± 2.43%]. The hydrogel via in-vivo experiments was demonstrated to greatly accelerate the repair of deep II degree burn wounds in rabbits when compared to the commercial trauma covering material (DUO DERM) and has strong promise for trauma repair (Fig. 8B). This highlights the significance of developing functional materials that mimic the structure of ECM for wound repair.

In addition, tissue-engineered artificial skin play influential roles in regulating the traumatic microenvironment. For instance, the FDA has approved StrataGraft, a new bilayer tissue-engineered skin composed mainly of mouse collagen, with top and bottom layers implanted with human keratinocyte and fibroblast, respectively, for the management of victims suffering deep second-degree burn wounds. By imitating the structure of the epidermis and dermis of human skin, it directly stimulates the natural healing of most wounds without the need for an additional repair [140]. This artificial skin is expected to revolutionize the future of clinical wound treatment by mimicking the epidermal and dermal structures of human skin to directly stimulate self-healing of most traumatic tissues without the need for autologous skin grafts.

### Self-healing wound dressing for motional wound

Motional wounds on stretchy parts have the increased wound dressing requirements of all wound types. Wounds on the neck or joint regions, for example, can be subjected to compression, tension and other stretchable pressure [8]. In these types of wounds, wound dressings are not only subjected to extra mechanical stress, but are prone to breakage or fracture. Breakage in dressings not only leads to disruption of their characteristics or even loss, but it also allows external microorganisms to invade the wound with infections [8]. Consequently, self-healing wound dressings have been proposed as a “smart” material that may fix functional and structural damage on its own [142, 143].

**Fig. 9:**
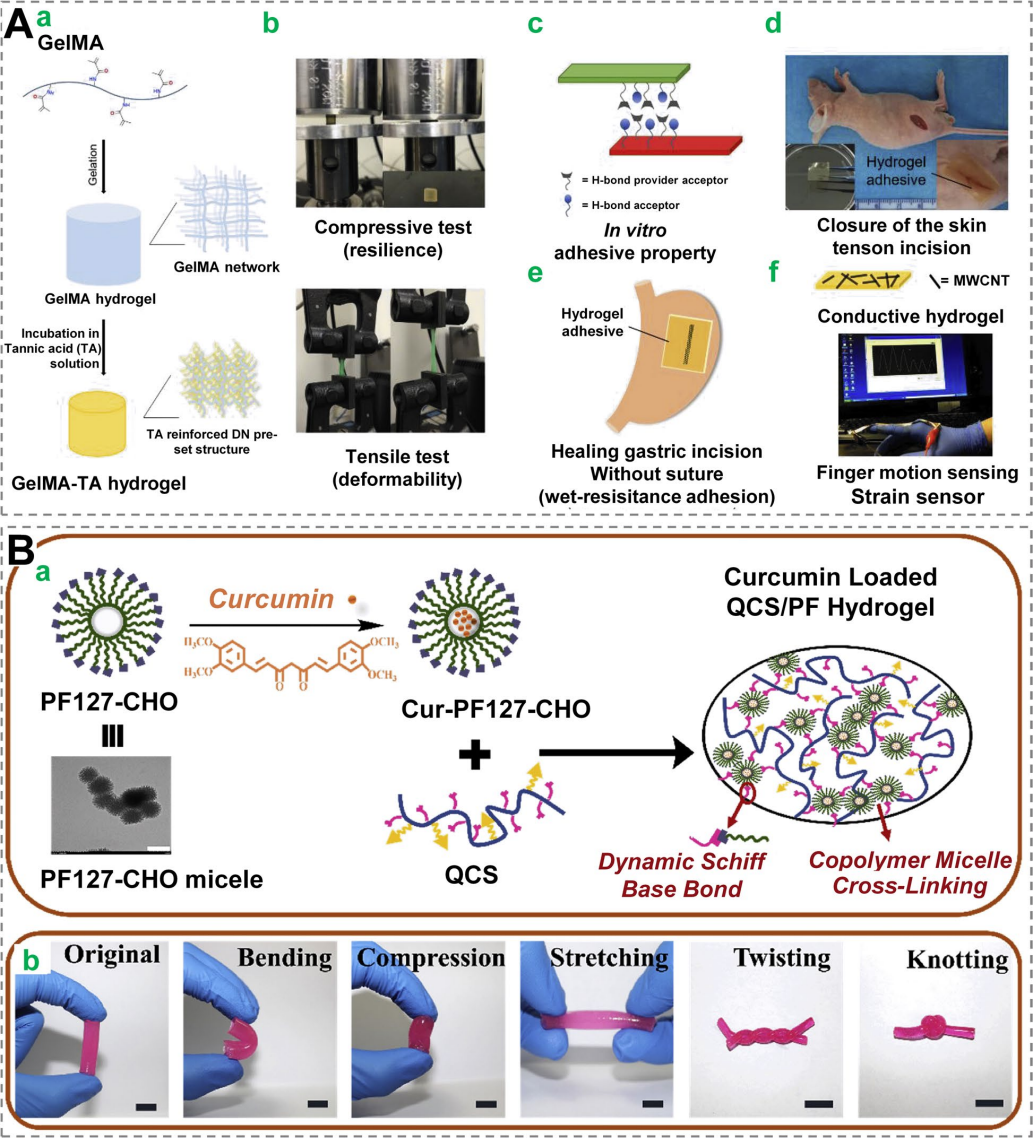
Self-healing wound dressings for motional wound. **A** Tannic acid (TA) was used as a multi-functional H-bond supplier to create a GelMA-based double-network (DN) hydrogel with versatile capabilities. The preparation of a versatile GelMA-TA hydrogel (a) with high stiffness, super-elasticity, deformability (b), and in vivo self-healing and adhesive properties is shown schematically (c). GelMA-TA gel has been used in biomedical applications such as skin wound closure (d), sutureless gastric surgery (e), and as a strain sensor when MWCNTs are present (f). Reprinted with permission from ref [69]. Copyright 2018 Elsevier. **B** Antibacterial adhesive injectable hydrogels with quick self-healing, extensibility, and compressibility were manufactured as wound dressing for joints skin wound healing by mixing quaternized chitosan (QCS) and benzaldehyde-terminated Pluronic®F127 (PF127-CHO) under physiological conditions. (a) Schematic presentation of the hydrogel and TEM images. Scale bar: 200 nm. (b) Rhodamine B coloured QCS/PF1.0 hydrogels in their natural bending, compression, stretching, twisting, and knotting shapes. Scale bar: 1 cm. Reprinted with permission from ref [146]. Copyright 2018 Elsevier

The constitutional dynamic chemistry technique, which involves the construction of a cross-linking network via dynamic and reversible chemical bonding, is mostly applied to manufacture self-healing dressings [144, 145]. Liu et al. designed a composite hydrogel (double-network) containing GelMA, as well as tannic acid whose dynamic hydrogen bonding aided this hydrogel to possess enhanced self-healing capacity (Fig. 9A) [69]. Qu et al. used the Schiff-base reaction to crosslink quaternized chitosan containing benzaldehyde-terminated Pluronic F127 to fabricate self-healing hydrogels for cutaneous wounds in joint regions [146]. This hydrogel has an excellent adhesive capacity and did not break or split when placed to the human elbow, even when flexed to a 120° angle (Fig. 9B). Besides, host–guest interactions between cyclodextrins and amantadine, NIPAM or silk fibroin have also been utilized to construct self-healing hydrogels [147, 148], mostly exhibiting substantial potentials for tissue repair. Taken together, although self-healing dressings have only been used to treat cutaneous wounds for less than a decade, they have received growing attention due to their unique self-healing, as well as good adhesive and mechanical properties [8]. In addition, further attention needs to be paid to how to better combine multiple functions and facilitate their translation to the clinic.

### Self-removal wound dressing

**Fig. 10:**
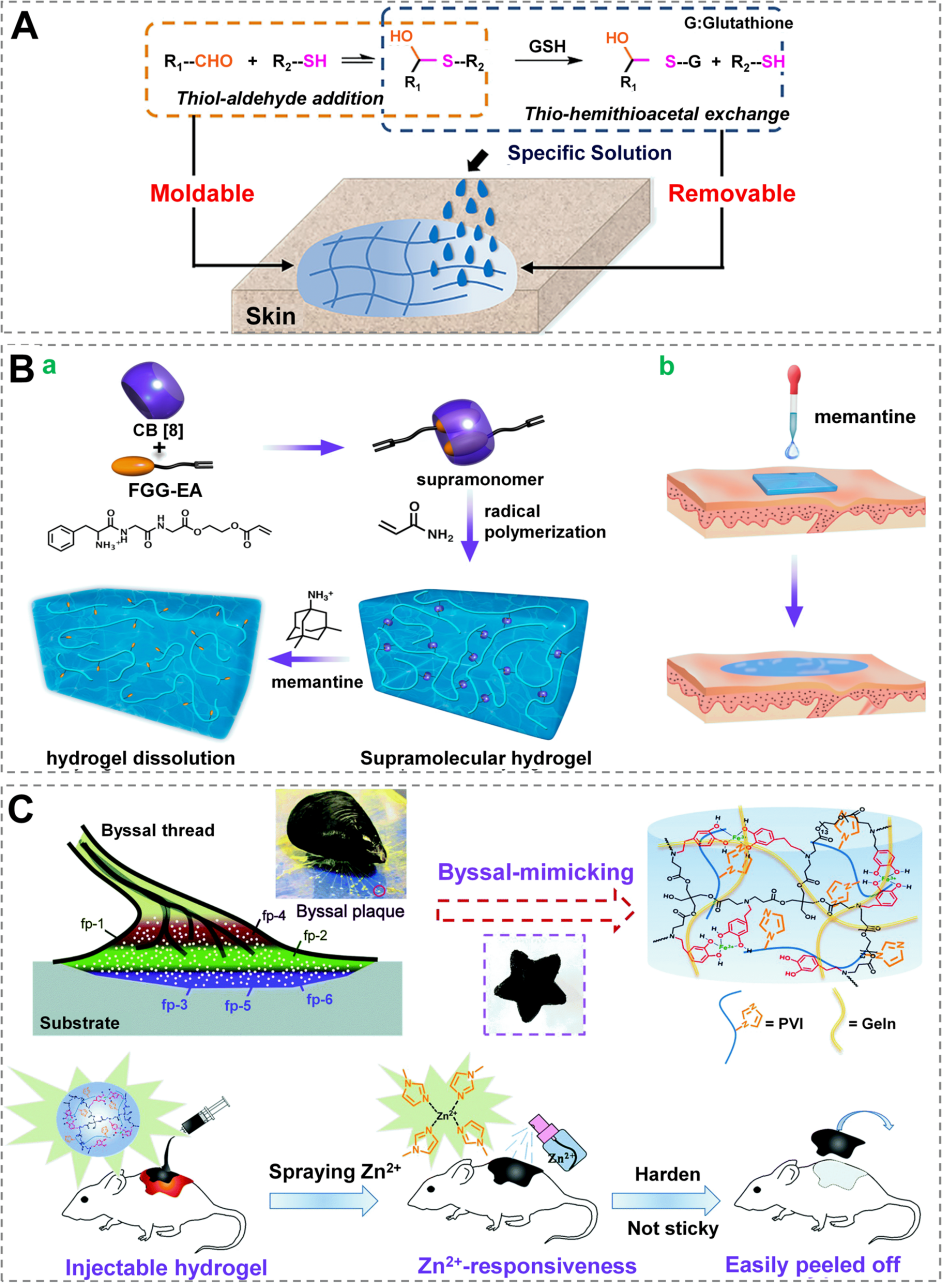
Self-removal wound dressings. **A** A reversible PEG-thiol-Aldehyde Addition Reaction and a Thiol-Hemithioacetal Exchange Reaction are used to make moldable and removable wound dressings, and a Thiol-Hemithioacetal Exchange Reaction is used to dissolve hydrogels. Reprinted with permission from ref [151]. Copyright 2019 American Chemical Society. **B** An easily removable wound dressing with supramolecular hydrogel. (a) Fabrication of supramolecular hydrogels from supramonomers and their breakdown under memantine irrigation and (b) its utilization as a wound dressing. Reprinted with permission from ref [152]. Copyright 2017 American Chemical Society. **C** A Michael addition reaction between dopamine, poly(ethylene glycol) diacrylate (PEGDA700), and pentaerythritol triacrylate was used to create an injectable adhesive hydrogel-based bandage (PETA). Spraying zinc ions on the dressing could quickly replace it. Reprinted with permission from ref [70]. Copyright 2020 Royal Society of Chemistry

Wound dressings’ changing may be painful and impair wound healing [149]. To overcome these shortcomings, self-removal dressing made using thermosensitive polymerization, dynamic thiol-aldehyde addition, light-triggered dissolution, ions addition (such as Zn^2+^), and other methods may be utilized. A hydrogel with self-removal capacity was designed using polyisocyanopeptide (a thermosensitive polymer). It retains in a gel state at room temperature but turns into liquid when cooled below 16 °C making disposal easier; nevertheless, this may limit its application in cold conditions [150]. To address this issue, PEG-thiols and oxidized dextran were combined to develop one hydrogel that underwent dynamic thiol-aldehyde addition between the aldehyde groups and the thiol, enabling this dressing that dissolved on demand (Fig. 10A) [151]. The thiol-hemithioacetal exchange reaction induced the hydrogel to disintegrate after the addition of free thiol molecules (such as glutathione or cysteine).

Light-triggered dissolution is another modality that enables self-removal property of wound dressings using non-invasive stimulus, which includes (but not limited to) NIR light and UV light [52]. Through host–guest noncovalent interaction between Cucurbit [8]uril (CB [8]) and the tripeptide Phe-Gly-Gly ester derivative (FGG-EA), Xu et al. [152] synthesized supramonomers which could be cross-linked by radical copolymerization of acrylamide to fabricate supramolecular hydrogels, as an easily removable wound dressing (Fig. 10B). The breakage of the hydrogen bond caused by increased temperature by NIR light helped the decomposition of the hydrogel, endowing it with self-removal property. Adding ions may also result in dressing dissolution, as demonstrated by Xie et al. [70]. An injectable dopamine-based adhesive hydrogel containing Poly(1-vinylimidazole) (PVI) was fabricated. However, its adhesive strength is rapidly reduced by spraying Zn^2+^ solution, which was attributed to the metal ion complexing established between PVI and Zn^2+^, reducing the hydrogel’s surface wettability (Fig. 10C). Notably, the metal biotoxicity of residual Zn^2+^ may limit its clinical application [75].

### Monitoring

Wound healing occurs in stages, each with molecular level changes which may disrupt the normal healing process. Refractory wounds can be caused by multiple factors, including infection, hyperglycemia and so forth [153]. Efforts have been made to design wounds dressings capable of monitoring the bacterial infection signs (such as temperature, pH, secreted enzymes or toxins) or other wound parameters (glucose level change, moisture level, oxygen concentration, etc. [4]).

**Fig. 11:**
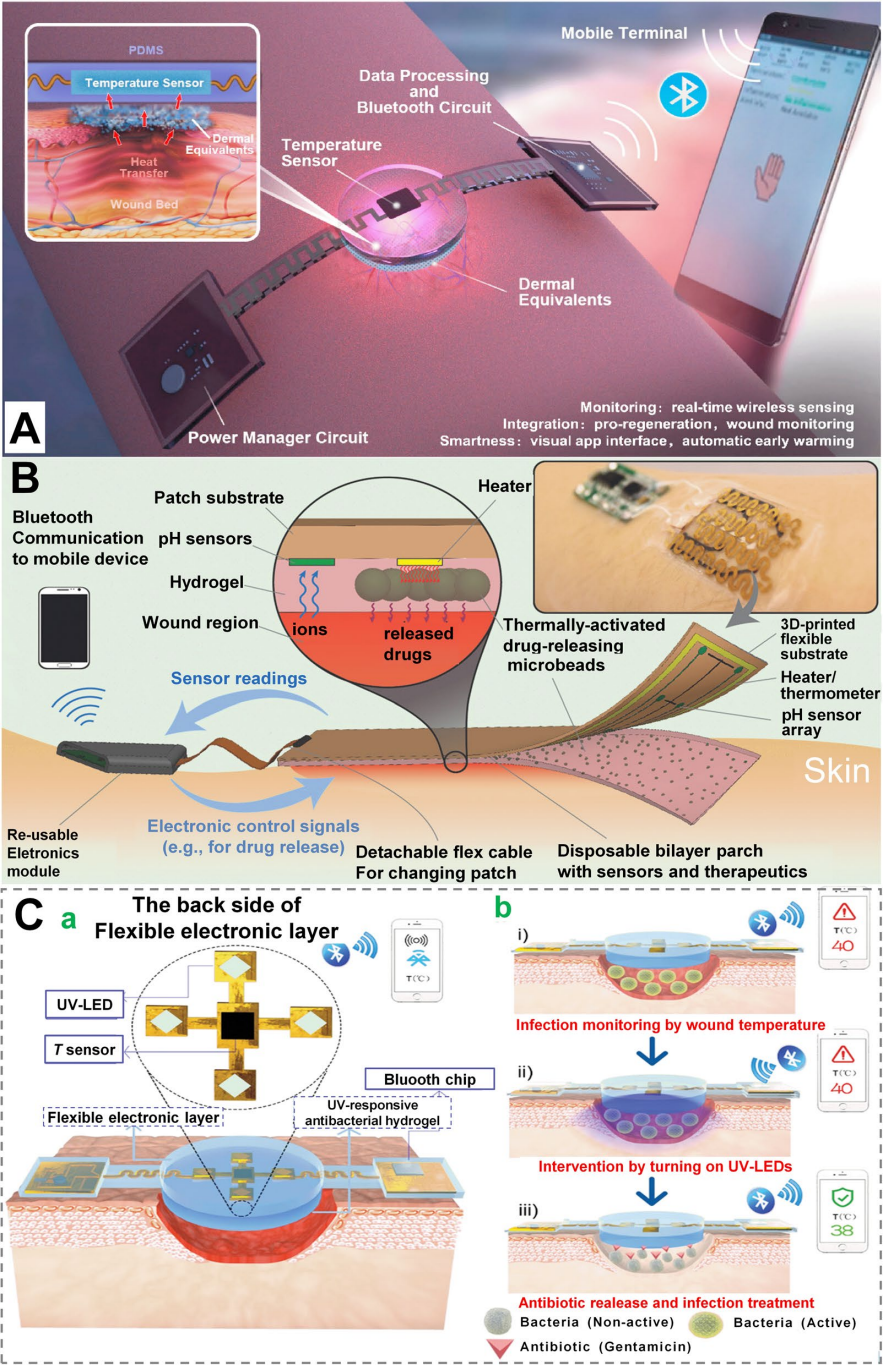
Wound dressings with monitoring function. **A** Temperature-monitoring wound dressing with temperature sensor, power manager circuit, data processing, and Bluetooth circuit that can send temperature changes to a mobile device in real time. Reprinted with permission from ref [158]. Copyright 2020 Elsevier. **B** Smart bandages with flexible pH sensors and a heater to trigger thermo-responsive medication carriers holding antibiotics for chronic wounds (wirelessly connected to smartphone). Reprinted with permission from ref [161]. Copyright 2018 Wiley-VCH. **C** Real-time monitoring and on-demand treatment of infected wounds using a smart flexible electronics-integrated wound dressing schematics and functioning principles. (a) Within a Bluetooth chip for wireless transmission, the integrated system contains a polydimethylsiloxane-encapsulated flexible electrical layer and a UV-responsive antibacterial hydrogel. (b) An illustration of the integrated system for monitoring infected wounds and providing on-demand therapy. Reprinted with permission from ref [71]. Copyright 2020 Wiley-VCH

The presence of alterations in the wound pH indicates the probability of bacterial infection. Based on this, He et al. fabricated a wound dressing possessing qualitative monitoring of infection via methylene blue, which turns colorless from blue on contact with bacteria [154]. To quantitatively monitor the infection level and its ongoing status, colorimetric method and electrochemical method were developed [155, 156]. By combining drug-containing scaffolds and color-altering pH sensors in alginate-based dressings, Mirani et al. fabricated a versatile wound dressing with mesoporous resin beads that were modified with pH-sensitive dye and then inserted in alginate for 3D printing. The color alteration of pH sensors was then shown on smart phone with a readable pH value [157]. Temperature, another indicator of infection, was monitored via a flexible chitosan-based dressing, consisting of a temperature sensor, microprocessors and bluetooth circuit that enabled wireless temperature display (Fig. 11A) [158].To monitor wound glucose concentration for efficient guidance of diabetic wound treatment, Jankowska et al. fabricated wound dressings capable of detecting pH and glucose concentrations in situ [159]. GOx was employed to convert glucose into a substrate for horseradish peroxidase, which oxidized fluorescent probes to turn an invisible change in wound status into a visible fluorescent signal [159]. Despite timely reporting of parameter changes at the wound site, these dressings still lack follow-up treatment outcomes for infection, diabetes, and other conditions.

Owing to advances of microelectronic technology and biomaterials in the past decades, smart wound dressings possessing real-time monitoring function have been fabricated, which may also release encapsulated drugs in situ on-demand to timely deliver therapeutics [160]. Mostafalu et al. firstly engineered a multilayer bandage equipped electrochemical pH sensors within the hydrogel layer, which also carried pNIPAM designed on a adaptable heater (Fig. 11B). In this device, a microcontroller linked to smartphones was connected by the heater and sensors. When the wound pH reached 6.5 after embedding the hydrogel within the substrate of temperature microsensor and parylene-based pH, this heater would automatically activate and begin the release of cefazolin from pNIPAM carrier [161]. Aside from smart, flexible and antimicrobial properties, a bi-layer wound dressing possessing on-demand drug delivery has been designed by Pang et al., enabling the early detection of bacterial infections (via temperature). Once an infection occurs, this UV-responsive hydrogel will be activated by UV light to release the loaded antibiotics into the wound bed in situ (Fig. 11C) [71]. Clearly, wound dressings are no longer limited to traditional single monitoring functions (pH, glucose, temperature, etc.), but to give appropriate interventions manually or automatically on the basis of real-time monitoring. Furthermore, this strategy can be better integrated with telemedicine, which will greatly reduce the differences between regions, benefiting both doctors and patients.

### Scar management

Patients with pathological scars suffer profound emotional, psychological, social and economic burdens [162]. Various approaches have emerged for scar therapy, such as agents, pressure therapy, laser/radiation therapy and surgery, as well as biomaterial-based dressings.

**Fig. 12:**
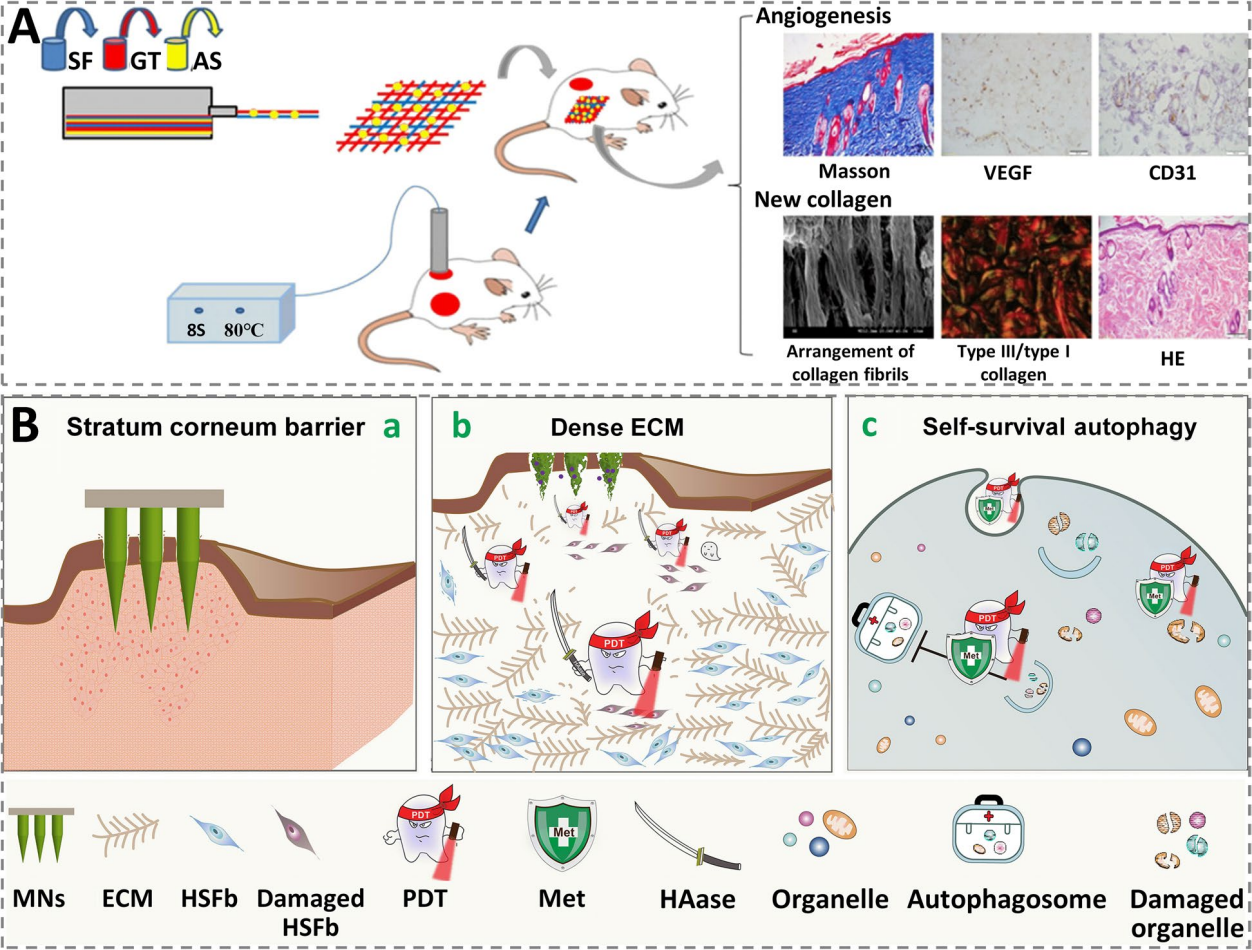
Wound dressings with scar management function. **A** Silk fibroin/gelatin (SF/GT) electrospun nanofibrous dressings loaded with astragaloside IV (AS) were created to stimulate wound closure, increase angiogenesis, regulate newly formed kinds of collagen, and improve collagen organization in burn wounds. Reprinted with permission from ref [166]. Copyright 2015 Elsevier. **B** Scheme of PDT with spear and shear, fully armed, for topical deep hypertrophic scar therapy. (a) Dissolving microneedles (MN) based on HAase penetrated the stratum corneum and delivered medicines into hypertrophic scar lesions; (b) By attacking the ECM, HAase-based MN worked as a spear to carry more ALA into deep lesions, enhancing the efficiency of PDT; (c) For increased PDT, Met was used as a shear to impede the self-survival autophagic process. Reprinted with permission from ref [72]. Copyright 2022 Elsevier

Although drug therapy (e.g., glucocorticoids) can improve scarring to some extent, wound dressings made of biomaterials have gotten considerable interest for scar management [4]. Pressure therapy (compression therapy) is an effective strategy used as HTS prevention and treatment in clinical, it usually entails elastic bandages constructed with elasticized fabrics or compression garments, which parallelize the collagen bundles by applying constant and uniform pressure to the wound [163]. Silicone gels are commonly utilized dressings for pathological scars in clinical, attributed to an occlusion substrate provided by gel sheeting to maintain the surroundings moist, enabling scar reduction [164]. Zhang et al. reported that combining a silicon stiffener layer with gradient studs and medical-grade silicone gel can provide a custom-made localized pressure and occlusion environment that enhances HTS treatment [164]. Other biomaterials, such as nanofiber, hydrogel, have also shown promise in scar treatment. Applying gelatin/polycaprolactone coated by dopamine, a nanofiber-based wound dressing was designed by Jiang et al. to inhibit post-operative defects caused electrospinning sutures [165]. Using a similar technology, silk fibroin/gelatin nanofibrous dressings were synthesized and functionalized by astragaloside IV (Fig. 12A). In-vivo experiments demonstrated the dressing fostered wound healing and minimized scarring [166].

With the rapid development of smart dressings, providing automated and precise treatment on the scar tissue in a controllable manner has drawn increasing attention [167]. Ghassemi et al. developed a smart wound dressing made of polycarbonate containing a wireless communication device, as well as a compression chamber equipped with a force sensor to achieve real-time pressure monitoring. The automated pressure delivery device was then linked to two full-thickness incisions on two pigs’ flanks made with an electric dermatome measuring 100 mm x 100 mm. This device can suppress the progression of HTS in ex vivo experiments by continually applying 30 mmHg pressure to wound scars for 2 weeks [168]. PDT mediated by 5-aminolevulinic acid (ALA) has emerged as a viable therapy for HTS. However, PDT’s efficiency has been limited by ALA’s weak penetration across biological barriers and fibroblasts’ pro-survival autophagy. To address this issue, Huang et al. constructed a soluble microneedle co-delivery system using the mechanically enhanced toughening and permeation-promoting effects of HAase as a “spear”, breaking through the stratum corneum and ECM’s barriers for drug delivery and achieving deep PDT for pathological scars. Besides, the autophagy modulator metformin (Met) served as a “shield” to prevent the “self-rescue” autophagy behavior of cells induced by PDT in order to minimize its resistance (Fig. 12B) [72]. In general, the treatment of scars is still dominated by traditional strategies, and automated and precise treatments via smart dressings are still in the early stage, with broad research and application prospects.

### Versatile biomaterials with Smart and/or responsive properties

Wound healing pathologies are mostly caused by a combination of anatomical or regional variables which contribute to prolonged ulcers or severe scarring. It is not surprising that a single component administered therapeutically has limited healing efficacy. Conversely, combined therapies including many ingredients cooperating in time and place to reestablish tissue function are desired [22]. As a more individualized approach, adapting these constituents to various wound types could lead to more satisfactory outcomes.

**Fig. 13:**
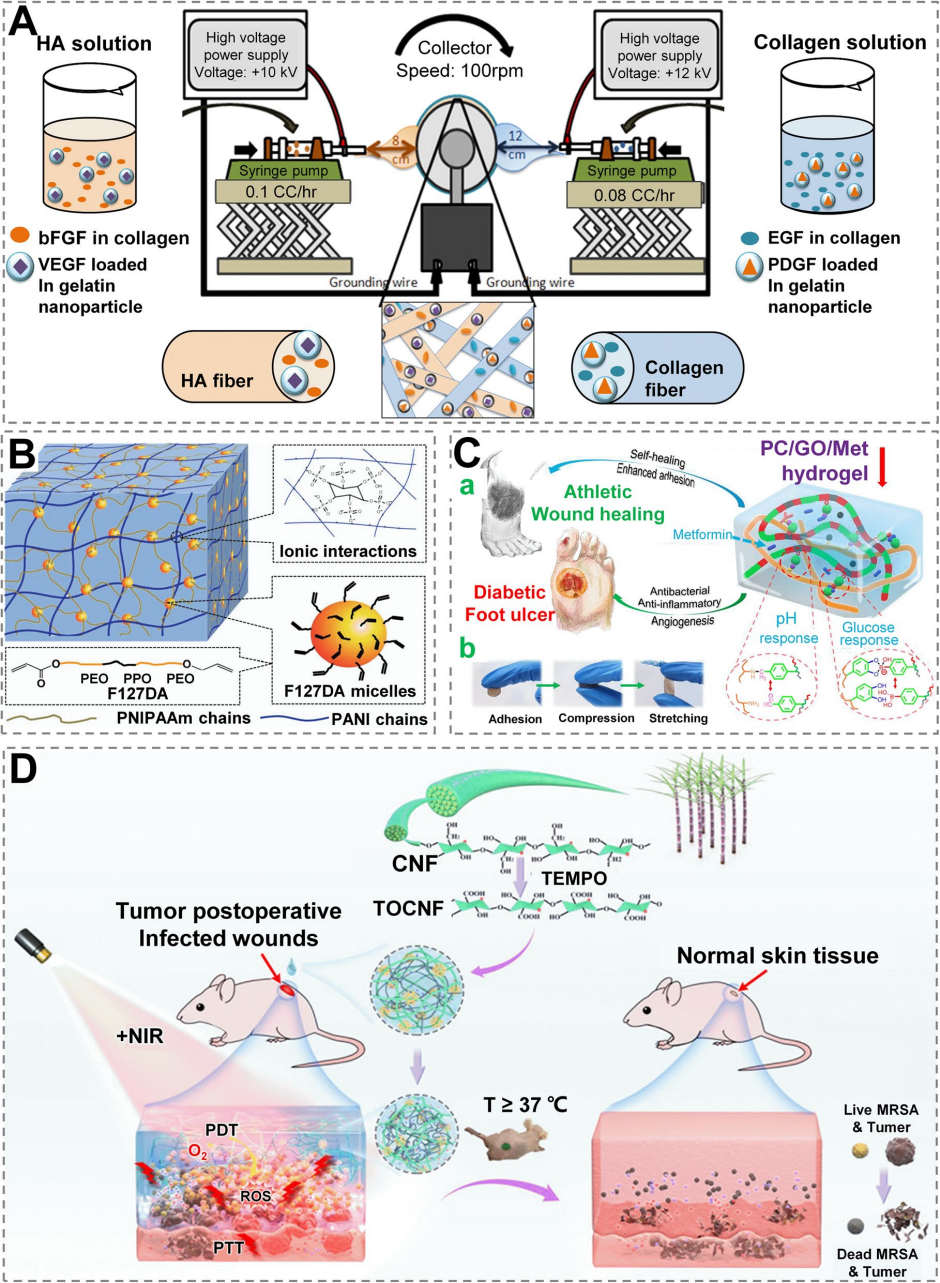
Versatile biomaterials with smart and/or responsive properties. **A** With the goal of repairing chronic wounds, four different growth factors (EGF, bFGF, PDGF, and VEGF) were loaded, either directly embedded in HA and collagen nanofibers or encapsulated in gelatin NPs (GNs) and subsequently incorporated into nanofibers. Reprinted with permission from ref [171]. Copyright 2014 Elsevier. **B** F-PNIPAAm/polyaniline (PANI) hydrogels have a network structure. PEO and PPO are poly(ethylene oxide) and poly(propylene oxide), respectively, in Pluronic F127 (F127DA). Reprinted with permission from ref [173]. Copyright 2018 American Chemical Society. **C** Schematic overview of structure and application of hydrogel dressings. (a) The structure, pH, and glucose response mechanism of PC hydrogel, as well as its application in diabetic foot ulcers and athletic wound healing, are depicted schematically. (b) Testing of mechanical properties of PC hydrogels. Reprinted with permission from ref [73]. Copyright 2022 American Chemical Society. **D** Possessing multiple responsive properties (temperature, pH and NIR) to release active drugs (like DOX), A pH-sensitive CNF grafted with terminated amino hyperbranched polyamines (HBP-NH2) as a substrate was used to develop a multi-functional and shape-adaptable liquid wound dressing (with CNF) for irregular tumor postoperative infected wounds, with ICG and PNIPAM loaded as NIR and temperature on/off buttons, respectively. Reprinted with permission from ref [174]. Copyright 2021 American Chemical Society

Microsphere-engineered skin containing EGF and mesenchymal stem cells promoted wound repair more effectively when compared to unloaded EGF-engineered mouse skin, accompanied by upregulation of granulation tissue, vascularity and regenerated sweat gland-like structures [169]. Compared with group control (cell-free intervention), radiation wounds intervened with MSCs transfected with β-defensin 3 and VEGF 165 healed faster and exhibited improved granulation tissues and collagen deposition [170]. Lai et al. created a multiple release system based on HA and collagen electrospun nanofibers containing gelatin nanocapsules. Within one month, this system could release various growth factors (bFGF, EGF, VEGF and PDGF) into wound bed in a controlled manner, which strongly enhanced the therapies (Fig. 13A) [171]. A novel technology termed nano-injection has recently been created, which is a hybridization of microneedle administration and localized electroporation in a pseudo-multi-electrode array form [172]. Based on an in-vitro CRISPR-Cas9 model, nano-injection showed great promise in wound repair, reflecting the advantages of excellent transfection efficiency and cell viability without cytotoxicity [22].

Notably, dual or multiple stimulus-responsive delivery systems, which are commonly loaded with one or more bioactive compounds for site- or spatiotemporally precise administration, are more cutting-edge and effective strategies. pH is commonly used in dual stimulus-responsive systems and is frequently paired with other stimuli (e.g., temperature, light, glucose, etc.) [52]. For instance, using PNIPAAm and polyaniline as the thermally responsive and the electrically conductive ingredients, respectively, Wang et al. developed a hydrogel (containing a dual network), whose advantages include suitable thermal reactivity, mechanical extensibility, and electrical conductivity (Fig. 13B) [173].

To establish a Met-sustained release system specifically for type II diabetic foot, Liang et al. designed a dual-responsive hydrogel dressing (stimulus: glucose & pH) with adhesive and self-healing capabilities (Fig. 13C), attributed to dual-dynamic bonding [73]. What’s more, the combination of graphene oxide and Met, as well as their synergistic effect, has been shown to improve wound repair in vivo [73]. Beyond this, a liquid wound dressing using cellulose nanofibril (CNF) that is multiple responsive (pH, temperature and NIR) and versatile (drug delivery, antibacterial ability, antitumor and biofilm-eliminating) has been fabricated to foster wound repair (Fig. 13D). In the study, pH-sensitive CNF grafted with end-amino hyperbranched polyamine was employed as the substrate, coupled with indocyanine green (ICG) and PNIPAM serving as the NIR light and temperature on/off switches, respectively [174]. Recently, PNIPAM/keratin dual network hydrogels with multiple sensitive properties (pH, ROS and temperature) were synthesized using ionic and covalent double cross-linking methods for the release of antimicrobial agents (i.e. chlorhexidine acetate), exerting self-stripping characteristics and facilitated skin regeneration [175]. Taken together, dual or multiple stimuli-responsive versatile biomaterials hold great promise for promoting wound healing and regeneration due to their ability to target multiple pathogenic factors or aspects.

## Clinical translation of smart biomaterial-based wound dressing

Despite advances in our understanding of wound repair and the emergence of new wound care products, obtaining the “perfect dressing” with effective clinical treatments remains a research priority. Gauze, as a conventional dressing, has significant drawbacks such as lacking elasticity, susceptibility to re-injury owing to dressing changes, etc [8]. In clinical practice, commercially available wounds dressings include films (like 3 M Tegaderm™, for first-degree burns or superficial lacerations), hydrogels (e.g., SilvaSorb, FlexiGel™, etc., for dry wounds), hydrocolloids, foams, alginates, hydrofibers, and skin regeneration materials (such as bi-layer dermal substitutes) [176]. Besides, foam-based dressings have been extensively employed in conjunction with NPWT to repair cutaneous wounds since the early 20th century [177]. Owing to the layer underneath (porous collagen-based scaffold) serving as dermal regenerate template, bi-layer dermal substitutes (including Lando®, Integra® and Pelnac®) have also been utilized to regenerate full-thickness skin defects [125].

With the increasing demands for speed, efficacy and operability in wound management, efforts are being made to innovate and improve existing skin repair materials, as documented in preclinical and clinical researches. For instance, a clinical trial of 40 subjects with NPWT revealed that both 125-mmHg polyurethane foam (high-pressure) and 75-mmHg silicone-covered dressing (low-pressure) successfully reduced the size of chronic wounds [177]. Cetinkalp et al. in a randomized trial demonstrated that a composite resveratrol-loaded bi-layered dermal matrix (Dermalix) contributed to a 2-fold faster healing rate of chronic wounds than standard dressings [178].

As for smart bio-responsive materials, the first responsive product is Visudyne® which was approved for ophthalmic disorders (pathologic myopia, ocular histoplasmosis and macular degeneration) via PDT, without inflicting additional organ damage, superior to the conventional laser therapies [179]. A previous review detailed several stimuli responsive drug delivery methods that have exhibiting promise potentials in preclinical or in-vitro research. However, only a handful of these systems have successful tendency to clinical trials, and those that are now being investigated are mostly for cancer treatment [180]. “TheraGauze” is a novel polymer dressing aimed at keeping the wound surface moist by selectively absorbing/providing moisture at specific sites [181]. Smart dressings have proven potential to be the next generation of wound dressings in terms of real-time wound healing monitoring and reporting, but none have yet reached the clinical trial stage. Therefore, it is necessary to establish standardized acceptable preclinical models to boost the development, approval and application of novel wound dressings through more extensive preclinical studies, which will benefit victims suffering skin and soft tissue defects.

## Perspectives and conclusion

Despite advances in our understanding regarding wound repair and the development of many wound care products, developing the “perfect dressing” with successful medical outcomes remains a focus of research [30]. Multiple studies have concentrated on the creation of responsive dressings. Nevertheless, it remains a challenge to get the responsiveness needed to deliver therapeutics to the right area, at the right time and at a physiologically meaningful dose. Hitherto, most of bio-responsive systems reported to date have not reach the above goals [52, 182]. When dealing with complicated microenvironments, smart systems that detect various stimuli would be advantageous in achieving on-demand release of certain medicines [183]. It is noteworthy that the ultimate goal of developing stimulus-responsive materials or systems is usually clinical translation benefiting patients [184]. However, the more sophisticated the material is designed to be, the more difficult it is to attain this goal. To sum up, the following are the top challenges that must be overcome before stimuli-responsive systems may be used in clinical settings: (1) Sensitivity and specificity: to minimize non-specific release in the absence of the correct signal and to optimize the trigger specificity of the dressing’s release in complex environments (e.g., chronic wounds). (2) Biocompatibility: to minimize side effects caused by original materials (such as cytotoxic and non-degradable PNIPAM polymer as thermo-responsive trigger.) and material forms (like nanomaterials spreading throughout the body and generating multisystem effects after entering the circulation). (3) Effectiveness: to endow the dressing with an on-demand function, or to minimize the interactions between different functions when designing multiple functions (such as drug delivery, monitoring, etc.). (4) Clinical translation: two key issues need to be addressed, namely the standardized and lengthy testing processes (involving tableside, preclinical, and clinical studies) before clinical applications, and the increasing complexity of material’s design reducing the possibility of scaled-up manufacture.

Wound management will shift significantly as medicine moves from a “one size fits all” approach to tailored therapies. Each form of wounds is unique, and the events that cause disruptions in the healing process vary in patients. Therefore, the development of smart systems automatically responding to pathological changes is expected to significantly boost the area, which might be accomplished in two ways: (1) To develop innovative smart materials that can react to the microenvironment of wounds and deliver the desired medications; (2) To develop smart systems capable of sensing pathological signals, analyzing data, and administering drugs automatically or under professional medical guidance. The former approach needs to concentrate on delivery strategies of smart systems, without damage to drugs’ activity and properties. The latter method will focus on the integration of diverse devices (such as biosensing, flexible electronics and wearable devices), which are closely correlated to wound monitoring and telemedicine.

To achieve more accurate on-demand control, the approach of combining different triggers using logic gates has been reported [185]. The DeForest group announced the first modular strategy utilizing a Boolean logic-based response for biomaterials, where three independent triggers (reductant, enzyme and light) were employed as inputs to the YES/OR/AND logic output, which could facilitate biomaterial degradation to release chemotherapeutic DOX. In this study, gel degradation was provoked by tumor pathophysiological environmental signals (increased MMPs) and “AND logic gating” was employed to control the release of DOX. However, exposure to MMPs alone did not trigger the release of chemotherapeutic agents. Likewise, logic gates have also been employed in other fields, such as diabetes monitoring and management [186, 187], a disease that is universally acknowledged to produce chronic wounds. Inspired by cancer and diabetes treatments, logic-gated reactive biomaterials may possess potentials to be applied in the management of cutaneous wounds. Equipping the biomaterial scaffolds with active or passive triggers and flexible YES/AND/OR logic gates may aid in achieving desired controlled release kinetics, which better match the natural processes of wound healing and regeneration.

In summary, the management of wounds caused by various skin disorders poses a huge health and financial burden on countries worldwide, while conventional dressings are still inadequate for refractory wounds or challenged by various deficiencies. There is no doubt that smart and versatile biomaterials will be the focus of future research and development, although there are still many obstacles on the “road” to their industrialization. We believe that with the extensive participation and persistence of global researchers to boost the area, the clinical translation of smart and versatile biomaterials will be soon realized for the benefit of patients.

## Data Availability

Please contact author for data requests.
